# Three Decades of Farmed Escapees in the Wild: A Spatio-Temporal Analysis of Atlantic Salmon Population Genetic Structure throughout Norway

**DOI:** 10.1371/journal.pone.0043129

**Published:** 2012-08-15

**Authors:** Kevin A. Glover, María Quintela, Vidar Wennevik, François Besnier, Anne G. E. Sørvik, Øystein Skaala

**Affiliations:** 1 Section of Population Genetics and Ecology, Institute of Marine Research, Bergen, Norway; 2 Dept of Animal Biology, Plant Biology and Ecology, University of A Coruña, Spain; University of Otago, New Zealand

## Abstract

Each year, hundreds of thousands of domesticated farmed Atlantic salmon escape into the wild. In Norway, which is the world’s largest commercial producer, many native Atlantic salmon populations have experienced large numbers of escapees on the spawning grounds for the past 15–30 years. In order to study the potential genetic impact, we conducted a spatio-temporal analysis of 3049 fish from 21 populations throughout Norway, sampled in the period 1970–2010. Based upon the analysis of 22 microsatellites, individual admixture, F_ST_ and increased allelic richness revealed temporal genetic changes in six of the populations. These changes were highly significant in four of them. For example, 76% and 100% of the fish comprising the contemporary samples for the rivers Vosso and Opo were excluded from their respective historical samples at *P* = 0.001. Based upon several genetic parameters, including simulations, genetic drift was excluded as the primary cause of the observed genetic changes. In the remaining 15 populations, some of which had also been exposed to high numbers of escapees, clear genetic changes were not detected. Significant population genetic structuring was observed among the 21 populations in the historical (global F_ST_ = 0.038) and contemporary data sets (global F_ST_ = 0.030), although significantly reduced with time (*P* = 0.008). This reduction was especially distinct when looking at the six populations displaying temporal changes (global F_ST_ dropped from 0.058 to 0.039, *P* = 0.006). We draw two main conclusions: 1. The majority of the historical population genetic structure throughout Norway still appears to be retained, suggesting a low to modest overall success of farmed escapees in the wild; 2. Genetic introgression of farmed escapees in native salmon populations has been strongly population-dependent, and it appears to be linked with the density of the native population.

## Introduction

Delineation of historical genetic structure can provide an insight into how contemporary evolutionary relationships among populations have been shaped by demographic, environmental and anthropogenic factors. Understanding these processes and their potential interactions will assist in predicting how natural populations are likely to evolve in relation to present and future challenges.

Salmonid fishes provide excellent opportunities to study evolutionary relationships among populations in both time and space. They inhabit a variety of habitats and display phenotypic and life-history variation among populations [1], some of which reflect local adaptations [1]–[3]. Furthermore, salmonids tend to exhibit highly distinct population genetic structuring, also in anadromous forms where high fidelity to natal stream (homing) serves to limit gene flow [4]. The Atlantic salmon (*Salmo salar*) is no exception to these characteristics, and the analysis of molecular genetic markers has revealed highly significant population genetic structuring throughout its entire range [5]–[8].

The contemporary population genetic structure of Atlantic salmon can be ascribed to a hierarchical system, whereby the largest genetic differences are observed among fish from different continents and regions [9]–[14]. These differences are to a large degree thought to reflect the patterns of post-glacial colonization. Within regions, highly significant genetic differentiation has been observed among salmon originating from different rivers [11], [15], [16], and in some cases, also between tributaries within the same river system [15], [17]–[19]. These differences, as revealed by molecular genetic markers, primarily reflect a combination of reproductive isolation and genetic drift, whereby demographics and landscape features play a modifying role [16], [17], [19]. Generally, where wild populations experience low human impacts, temporal genetic stability has been reported [20], [21].

Atlantic salmon populations have been heavily exploited and influenced by a wide-range of anthropogenic factors over a long period of time [22]. Adding to the list of challenges since the 1970’s, is the hundreds of thousands of domesticated salmon that escape from farms on a yearly basis, which display a wide range of interactions with wild conspecifics [23]. Although escapees display high mortality post-escapement [24], [25], they have been recorded in rivers throughout the species’ native range, such as England [26], Scotland [27], [28], North America [29], and Norway [30]. Escapees have also been observed in rivers located in countries where salmon farming is not practiced [29].

Genetic changes in native Atlantic salmon populations as a result of introgression from farmed escapees have been observed in Ireland [31]–[34] and North America [35]. Looking beyond these studies that have been conducted in single rivers, an analysis of seven Norwegian Atlantic salmon populations revealed significant changes in several rivers that had displayed large numbers of farmed escapees on the spawning grounds [36]. However, although farmed escapees have been observed in natural populations for over three decades, and in many regions these numbers exceed wild spawner abundance, the impact this has had on population genetic structure remains elusive. It is therefore not surprising that there are global concerns regarding the genetic integrity of wild populations [23], [37]–[41].

Norway is the world’s largest commercial producer of Atlantic salmon, and is the country where the highest numbers of farmed escapees have been recorded on the spawning grounds. Therefore, Norway represents an ideal country in which to examine how genetic structure has changed both within and among native Atlantic salmon populations in response to widespread migration of farmed escapees onto the spawning grounds. Here, we have conducted a spatio-temporal genetic analysis in order to investigate the potential genetic impacts of farmed escapees on population structure throughout an entire country.

## Materials and Methods

### Study Design

Atlantic salmon farming in Norway is currently based upon rearing multiple domesticated strains and sub-strains that were initially founded on fish originating from over 40 Norwegian rivers in the 1970’s [42]. Thus, while the allele frequencies of the farmed strains are generally distinct to each other due to founder effects [43], they overlap with the allele frequencies of Norwegian wild populations [43], [44]. Over time, farmed escapees do not originate from a single farmed strain, but from multiple strains. The result of this is that the gene flow signal from escapees represents a dynamic mixture of allele frequencies. Thus, the detection of genetic changes in wild populations when gene flow comes from multiple farmed sources is far more complicated [45] than where a set of populations are supplemented by a single and readily defined hatchery source [32], [46]. In the latter case, it is straight-forward to demonstrate that the allele frequencies in the recipient wild population converges with the allele frequencies with its donor. However, for the case of multiple farmed strains, the recipient wild population will not converge with any given farmed strain over time, and genetic introgression may be partially concealed [45].

Increasing the complexity of detecting genetic introgression of farmed escapees in wild Atlantic salmon populations is that the farmed strains (and therefore their allele frequencies) have, and continue to change significantly with time, i.e., some of the populations used at an earlier stage have been terminated or combined with other strains, while new sub-strains (e.g., in response to QTL selection) have been established. Consequently, it is not possible to accurately reconstruct the allele frequencies of the farmed escapees in Norway over the 15–30 year period in which this study is conducted. Nevertheless, despite the above challenges, modeling has demonstrated that gene flow from farmed escapees will lead to a reduction in genetic structure among wild populations [45], [47]. This is because over time, wild populations will be exposed to the average allele frequency from the major strains, and this will start to erode the existing allele frequency differences among wild populations. Furthermore, modeling has shown that genetic changes in wild populations as a result of farmed escapees spawning may be detected, although its likely to be underestimated [45].

As a consequence of the situation described above, the methodological approach implemented in this study is to look at both within and among-population genetic structure in the time-period where the numbers of escapees reported in Norwegian rivers has been highest (i.e., the last 15–30 years). Have native Norwegian salmon populations displayed temporal genetic changes in this period? And if so, can genetic drift be excluded as the primary driver of these temporal changes? Furthermore, where temporal genetic changes have been observed, have the populations become more similar or more differentiated to each other?

### Biological Samples

**Figure 1:**
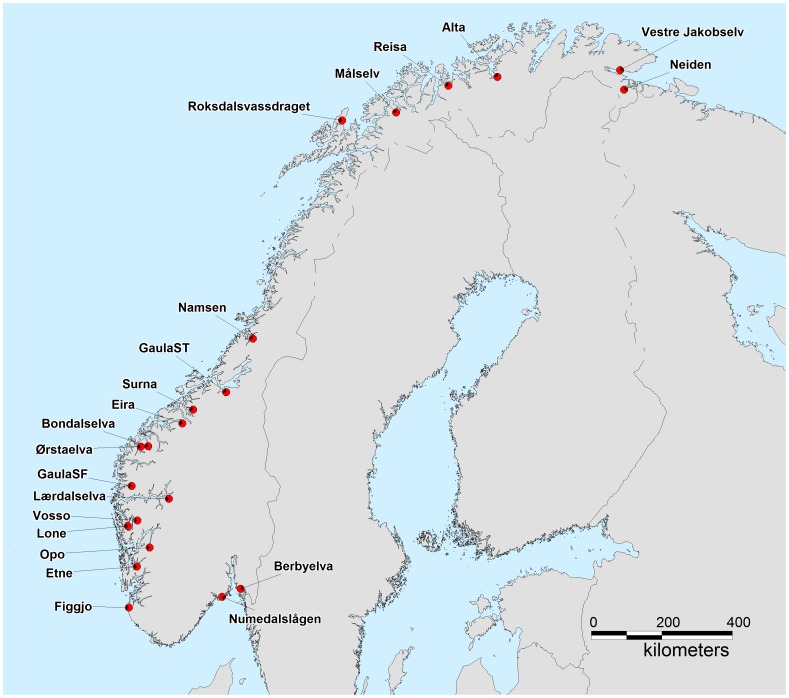
Norwegian rivers where historical and contemporary samples of Atlantic salmon populations were collected for the present study.

Historical and contemporary samples of Atlantic salmon populations were collected from 21 rivers spanning the entire Norwegian coastline which extends over 2500 km (Fig. 1; Table 1, 2). Populations were chosen primarily due to the availability of archived scale samples which were essential to re-construct the historical baseline (pre- or early aquaculture industry), and, availability of contemporary samples (year 2000+).

**Table 1:** Numbers and types of samples collected from 21 Atlantic salmon rivers.

Population	Sample size (n)	Sample type (NSR)	Population	Sample size (n)	Sample type (NSR)
Neiden H (1979–82)	79	SP (1)	GaulaSF H (1987–93)	40	SP (1)
Neiden I (1989–93)	43	SP	GaulaSF C (2006–08)	83	SP
Neiden C (2009)	93	SP	Lærdalselva H (1973)	95	SP (1)
V. Jakobselva H (1989–91)	96	SP (1)	Lærdalselva I (1996–97)	65	?
V. Jakobselva C (2007–08)	101	SP	Lærdalselva C (2005–08)	53	SP
Alta H (1988–90)	39	SP (1)	Vosso H (1980)	49	SP (1)
Alta C (2005–2007)	85	P	Vosso I1 (1993–96)	66	SP
Reisa H (1986–91)	48	SP (1)	Vosso I2 (2007–08)	48	SM
Reisa C (2006)	61	P	Vosso C (2008)	42	SP
Målselva H (1986–88)	47	SP (1)	Loneelva H (1986–93)	60	SP (0)
Målselva C (2008)	30	P	Loneelva C (2001–07)	52	SP
Roksdalsvassdraget H (1987–93)	37	SP (1)	Opo H (1971–73)	54	SP (0)
Roksdalsvassdraget C (2008)	94	SP	Opo I (2000)	46	P
Namsen H (1977)	92	SP (1)	Opo C (2010)	60	P
Namsen I (2000)	58	SP	Etne H (1983)	88	SP (1)
Namsen C (2008)	102	SP	Etne I (1997–98)	76	P
GaulaST H (1986–94)	48	SP (1)	Etne C (2006–2008)	88	SP
GaulaST C (2006–08)	106	SP	Figgjo H (1972–75)	57	SP (1)
Surna H (1986–89)	30	SP (1)	Figgjo I (1987–90)	41	SP
Surna C (2005–08)	52	SP	Figgjo C (2006)	72	SP
Eira H (1986–94)	34	SP (0)	Numedalslågen H (1989–93)	43	SP (1)
Eira C (2005–2008)	50	SP	Numedalslågen C (2007–08)	72	SP
Bondalselva H (1986–88)	44	SP (0)	Berbyelva H (1988–93)	46	SP (1)
Bondalselva C (2007)	16	P	Berbyelva C (2007–08)	94	SP
Ørsta H (1986–89)	40	SP (1)			
Ørsta C (2006–08)	34	SP			

Population = name of river with postscript letter H = historical sample, I = intermediate sample, C = contemporary sample. Life stage sampled = SP = spawners, E = eggs, A = alevins, F = fry, P = parr, SM = smolt, NSR = National Salmon River (river protected by extra legislation from government): 1 = yes, 0 = no.

**Table 2:** Characteristics of the rivers including catch statistics and numbers of escapees.

Population		Farmed escapees in the river			River characteristics							
		Yearscounted	Unweightedmean* (Range)	Weightedmean**	Local stocking?	2010catch (kg)	2010catch (n)	1990catch (kg)	1990catch (n)	Anadromousarea (km^2^)	Conservation attainment(2007–2010)	
Neiden		1	12%	2%	No	4.907**	1390	7099	NA	21.4	98%	
V. Jakobselv		18	30% (3–65)	20%	No	7.127	2283	1008	272	15.4	322%	
Alta		15	6% (0–22)	5%	M(S)	15.865	3403	9959	1953	57.0	228%	
Reisa		12	31% (0–100)	5%	L	7.280	1324	3044	585	53.0	177%	
Målselv		15	16% (4–36)	8%	L	11.614	2362	4992	908	20.0	249%	
Roksdalsvass.		19	7% (0–47)	3%	No	1.317	556	NA	NA	3.3	130%	
Namsen		21	27% (10–59)	11%	L(A)	20.360	4818	32075	8019	190.7	188%	
GaulaST		16	6% (0–22)	4%	M(A+E)	32.721	5690	25068	5334	93.6	224%	
Surna		7	28% (0–56)	14%	H(S+F)	7.320	1364	7750	2348	35.1	136%	
Eira		7	16% (0–44)	17%	H(S+P)	2206	549	580	NA	7.0	119%	
Bondalselva		10	27% (0–83)	17%	L(A)	521	175	7500	2143	2.1	124%	
Ørstaelva		15	41% (8–78)	22%	M(A)	1.375	502	4040	1616	4.9	60%	
GaulaSF		13	31% (4–65)	17%	M(A+E)	891	300	2071	628	10.5	144%	
Lærdalselva		4	2% (0–2)	4%	H(F)	Banned*	NA	4371	599	18.2	NA	
Vosso		14	45% (0–71)	29%	H(S+P)	Banned***	NA	880	91	15.3	NA	
Loneelva		16	8% (0–26)	7%	M(A+F)	244	107	363	214	0.4	133%	
Opo		2	50% (0–100)	89%	L(F+S)	Banned***	NA	612	146	5.8	NA	
Etne		19	57% (0–100)	35%	L(E+S)	Banned***	NA	7778	2431	3.7	156%	
Figgjo		14	9% (0–28)	9%	L(A+E)	4393	1466	7326	3330	5.4	175%	
Numedalslågen		15	7% (0–50)	5%	L(A)	7.729	1695	8791	2442	79.4	93%	
Berbyelva		6	4% (0–11)	2%	L	1134	181	304	74	3.3	582%	

Years counted = numbers of years in which farmed salmon were counted in the river, % of farmed salmon = the mean percent of farmed salmon observed in these populations based upon the unweighted mean = average percentage of farmed salmon in spawning population in the period 1989–2009 [50], [51], weighed mean = weighted average percentage of farmed salmon in the population combining data from both sports-fishing and spawning population samples [52]; range for the unweighted mean refers to the lowest and maximum percentages of farmed salmon observed in the spawning populations (this also includes recordings with very low numbers of observations in some years [49]). Local stocking history and river catch in 2010 statistics Norway www.ssb.no, and 1990 [125];

Na = not available.

* = treated against *Gyrodactylus salaris*;

** = Norwegian zone;

*** = population collapse or strongly reduced;

smolt and parr stocking activity: <5000 : Low; 5–15000: Medium; >15000: High (eggs, alevins and fry converted to smolt numbers by calculating 10% survival); anadromous area available to smolts [49], and conservation attainment which is the average attainment of the conservation limit for each specific river as defined by the numbers of female salmon left in the river after fishing mortality in relation to the number of eggs required to achieve the rivers estimated carrying capacity [49].

Historical samples were exclusively represented by fish scales taken from adult spawners captured in their specific rivers by rod and line (Table 1). Intermediate (neither the oldest nor newest set of samples from any given river system), and contemporary samples, were mostly represented by scale samples taken from adult fish captured by rod and line fishing or various research projects. Therefore, no specific licenses were applied for nor required to collect these samples for this study. Prior to any genetic analysis, all scale samples were analysed for growth patterns in order to exclude any salmon that had directly escaped from fish farms [48]. For some of the intermediate and contemporary samples, adult spawners were not available (for example due to closure of rod and line fishery). Instead, samples of juvenile fish were included for these populations. The historical samples were not collected from the exact same time period (Table S1), however, this was factored into some of the analyses.

Some of the relevant available information for the populations included in this study, which can be found in Norwegian reports [49]–[52] have been placed into Table 2. Importantly, this information includes the frequency of farmed salmon that have been observed in these populations in the period 1989–2009. Observations of farmed escaped salmon in Norwegian populations are primarily recorded by two approaches. One of the methods is based upon the percent of farmed fish in the angling catch during the summer sports fishing season, while the other is based upon the percent of farmed fish observed during dedicated autumn (spawning site) surveys. As farmed salmon tend to migrate later than wild salmon into freshwater [30], the autumn surveys tend to show higher percentages of farmed fish. However, the surveys of farmed fish frequency in the autumn usually involve sample sizes smaller than the summer angling catch surveys, are conducted less frequently, and are conducted in fewer rivers [49]. Nevertheless, the potential for genetic interaction is more tightly linked to the frequency of escapees observed on the spawning sites during the autumn than found in the summer angling catches. Therefore, we have chosen to use both estimates in the present study. First we use the un-weighted mean percent of farmed fish observed in the spawning surveys (i.e., averaging the percent farmed fish observed for the number of years in which they survey was conducted), in addition to using a weighted average based upon combining both summer sports fishing and autumn survey data that has been recently used to categorise over 100 Norwegian rivers in their degree of potential influence from farmed escaped salmon [52]. These estimates have then been compared with the temporal genetic changes observed for each river by regression analysis.

Samples of farmed salmon have been included for the analysis of admixture. These samples were selected from multiple data sets that have been analysed to identify the farms of origin for escapees as a DNA forensic service for the Norwegian ministry of fisheries in the period 2006 - present [53]–[57]. A total of nine farm samples, each of approximately 45 fish, were chosen based upon their large genetic differences to each other, and, in order to represent some of the genetic diversity found among salmon farms and farmed strains in Norway.

### Genotyping

DNA extraction was performed in 96-well plates using the Qiagen DNeasy®96 Blood & Tissue Kit. Each DNA plate contained two or more negative controls.

The following twenty two microsatellite loci were used; *SSsp3016* (Genbank no. AY372820), *SSsp2210*, *SSspG7*, *SSsp2201*, *SSsp1605*, *SSsp2216* [58], *Ssa197*, *Ssa171*, *Ssa202* [59], *SsaD157*, *SsaD486*, *SsaD144* [60], *Ssa289*, *Ssa14* [61], *SsaF43* [62], *SsaOsl85* [63], *MHC I* [64] *MHC II* [65], *Ssa19NVH* (Genbank no. AF256670), *CA060208* [66], *SsalR002TKU* and *SsalR010TKU* [67]. Amplifications were conducted in four multiplex reactions (conditions available from the authors). PCR products were analysed on an ABI 3730 Genetic Analyser and sized by a 500LIZ™ size-standard. Automatically binned alleles were manually checked by two researchers prior to exporting data for statistical analyses.

Microsatellites are known to be prone to genotyping errors [68], [69], even under strict protocols [70]. Eighteen of the microsatellite markers implemented here are routinely genotyped at IMR, and have revealed low error rates [55]. Within the present data set, some samples were re-analysed in order to increase the genotyping coverage and provide an ad-hoc quantification of genotyping quality.

### Statistical Analyses

For most of the statistical analyses conducted, samples were grouped into historical, intermediate and contemporary data sets. Other sub-sets of the data set were analysed for specific tests (i.e., including reduced sets of populations and markers). These variations are identified in the results. Bonferroni adjustment of the significance level for multiple testing was not presented. Instead, statistical significance was tested at α 0.05 and a more stringent level of α 0.001.

The genotype distribution of each locus in each population was compared with the expected Hardy-Weinberg distribution using the program GenePop [71] as was the linkage disequilibrium. Both were examined using the following Markov chain parameters: 10000 steps of dememorisation, 1000 batches and 10000 iterations per batch. Relative genetic variation in each population was assessed using allele frequency data from which observed heterozygosity *Ho*, expected heterozygosity *He*, allelic richness, F_IS_ and pairwise F_ST_ were calculated using MSA 4.05 [72].

In order to test whether the global F_ST_ among historical populations was significantly larger than the global F_ST_ among contemporary populations, a bootstrap test based on 10 000 re-sampled datasets was computed. For each resample, the global F_ST_ in historical and contemporary data was calculated based on a random sample of 30% of the individuals from each population and 30% of the markers (7 out of 22). After re-sampling, the distribution of the 10 000 differences between historical and contemporary F_ST_ was used to test the alternative hypothesis (H_1_: F_ST_ historical > F_ST_ contemporary) against the null hypothesis (H_0_: F_ST_ historical ≤ F_ST_ contemporary).

The program Geneclass 2.0 [73] was used to perform genetic assignment. First, the program was used to conduct self-assignment among the 21 populations in the historical and contemporary data sets. Thereafter, the historical genetic profile for each population was used as the baseline, while individual fish representing the contemporary sample for each population was assigned to their respective baseline population. Exclusion was assessed at a significance level of α 0.001 using all 22 loci, and the reduced set of 14 loci, with the Rannala & Mountain simulation method [74].

In order to investigate the potential relationship between geographic and genetic distance (F_ST_) in the historic and contemporary data sets, Mantel tests were conducted with the software PASSaGE [75] and significance was tested after 10 000 permutations. Genetic differentiation among populations was estimated by the Analysis of Molecular Variance, AMOVA [76] implemented in the program Arlequin [77], and significance was based upon 10 000 permutations.

A growing number of statistical approaches are available to identify putative non-neutral loci [78]. First, we used a hierarchical Bayesian method [79] as implemented in BayeScan software [80]. Secondly, we used the Fdist approach [81], implemented in LOSITAN [82] selection detection workbench for codominant markers. As a result, a subset of fourteen neutral microsatellite loci was obtained. Full details and results of these analyses are available in Text S1.

To investigate population structure we identified genetic clusters in the total and neutral dataset with the Bayesian model-based clustering algorithms implemented in STRUCTURE v. 2.3.3 [83]–[85] under a model assuming admixture and correlated allele frequencies without using population information. Five to ten runs with a burn-in period of 50000–100000 replications and a run length of 500000–1000000 Markov chain Monte Carlo (MCMC) iterations were performed for a variable number of clusters (see footnotes of corresponding barplots for more detailed information). We then applied an ad hoc summary statistic ΔK which is based on the rate of change of the ‘estimated likelihood’ between successive K values [86]. When needed, runs of the selected K were averaged with CLUMPP version 1.1.1 [87] using the LargeKGreedy algorithm and the G’ pairwise matrix similarity statistics and results were visualized as a barplot. Admixture analyses were conducted both with wild salmon and with a combination of wild and farmed salmon (see results).

Genetic drift may be considered as a random evolutionary process whereby a population’s allele frequency at one or more loci can change through time. This process is especially influential in small populations [88], [89]. Thus, in order to evaluate whether any of the populations included in the present study were very small and likely to be strongly influenced by genetic drift, the effective population size (*Ne*) was computed in each river. This was conducted separately for both the historical and contemporary samples, using the one sample linkage disequilibrium method implemented in the program LDNE [90]. Furthermore, in order to investigate the plausibility that genetic drift could have been the primary driver of the temporal genetic changes observed in some of the populations studied (see results), we simulated genetic drift on these historical populations. For these computations, a methodological approach inspired by an available software for simulating genetic drift [91] was implemented in R (R Development Core Team). Starting from the observed historical sample, additional generations were simulated by gene dropping, so that every additional generation were obtained from the previous one assuming random mating, equal sex proportions, no migration, selection nor migration. Drift was thus assumed to be the only evolutionary force acting upon the populations and markers were unlinked. In order to investigate how *Ne* influences genetic drift over multiple loci simultaneously, these simulations were conducted 1000 times for each population assuming *Ne* of 25, 50, 75, 100, 200, 300, 400 and 500, and setting a non-overlapping generation interval to 5 years. The number of generations in which drift was simulated was thereafter a function of the number of years between the historical sample and the corresponding contemporary one, divided by 5, and then rounded up to the nearest whole generation. The genetic distance (F_ST_) between the observed historical genetic profile for that population, and the 1000 simulated contemporary populations at each level of *Ne*, were then compared to the genetic distance that was actually observed between the historical and contemporary sample. The probability that the observed pair-wise F_ST_ was greater than the genetic drift simulated F_ST_ was thereafter computed. As in [91], this was achieved by comparing the proportion of the observed F_ST_ values exceeding the genetic-drift simulated F_ST_ values for that population. These simulations were also used to look at global F_ST_ values, and evaluate allelic richness in the presence of genetic drift.

## Results

### Genotyping Quality

The final data set consisted of 3049 salmon displaying a mean genotyping coverage of 96.1%. Coverage ranged from 87.1% for the marker Ssa*D157*, to 99.4% for the marker *SsaF43*. When genotyping success was broken down into the historical and contemporary data sets, coverage was 94.8% and 97.9% respectively.

From 9314 alleles scored independently on two occasions, a mean genotyping error rate (defined here as inconsistent scoring between two independent runs of the same sample) of 0.1% was computed. The absolute number of alleles scored twice/errors observed = 7506/7, 806/1, and 1002/2 for the historical, intermediate and contemporary samples respectively. This is consistent with previous estimates for these [55] and other genetic markers [70], [92] in this laboratory. Allelic distribution in the historical and contemporary data sets (pooled populations) did not reveal a disproportionate loss of the large alleles in the historical samples (Table S2).

### HWE, LD and Potential Neutrality of Markers

Analysis of HWE and LD can identify technical issues (marker robustness and genetic linkage between loci) and biological processes (mixing of populations and population disturbance through introgression). At the significance level of α 0.05, a total of 32 (7.1%), 5 (2.9%) and 32 (7.2%) loci by sample combinations displayed significant deviations from HWE in the historical, intermediate, and contemporary samples respectively (Table 3; Table S3–supporting information). At α 0.001, the number of deviations dropped to 2, 1, and 1 in the three data sets respectively. No more than 4 of the 21 populations deviated for any given locus in any of the three data sets demonstrating once again that the markers were of high technical quality. Excluding the historical sample for Vestre Jacobselv, where 9 loci departed from equilibrium at α 0.05 (one of which remained significant at α 0.001), deviations from HWE were distributed among the rivers, with most displaying deviations in 0–3 loci at α 0.05 (Table 3; Table S3).

**Table 3:** Effective population size, within-sample genetic diversity estimates, and temporal genetic stability between historical and contemporary samples within 21 Atlantic salmon rivers located throughout Norway. For full data, including locus specific statistics see Table S2.

Rivers	Within-sample diversity								Temporal stability				
	Historical				Contemporary				F_ST_ historical vs. contemporary		Exclusion from hist. <0.001		Temporalchange?
	LD	HW	A_R_	*Ne* (95% CI)	LD	HW	A_R_	*Ne* (95% CI)	22 loci	14 loci	*22 loci*	14 loci	
Neiden	22	0	201	430 (296–760)	7	1	203	Inf (3179-Inf)	0.0009	0.0011	6%	3%	No
V. Jakobselv	85	9	190	79 (71–91)	32	0	200	169 (148–196)	0.0064**	0.0076**	16%	7%	Yes
Alta	5	2	187	Inf (990-Inf)	13	1	190	4860 (856-Inf)	−0.0002	0.0010	2%	1%	No
Reisa	11	2	185	272 (180–533)	61	1	179	80 (69–94)	0.0041*	0.0020	15%	10%	No
Målselv	10	2	199	Inf (−1361-Inf)	3	0	207	411332# (322-Inf)	−0.0026	−0.0011	13%	7%	No
Roksdalsvass.	9	0	205	516 (241-Inf)	66	2	206	384 (291–554)	0.0014	0.0023	20%	12%	No
Namsen	10	0	208	3526 (835-Inf)	14	1	209	914 (549–2550)	0.0013*	−0.0012	9%	3%	No
GaulaST	4	0	206	Inf (2162-Inf)	10	1	208	24753 (1358-Inf)	0.0012	0.0018	12%	14%	No
Surna	9	0	203	1530# (252-Inf)	11	1	216	Inf (965-Inf)	0.0025	0.0035	34%	17%	No
Eira	11	2	209	378 (196–3201)	11	0	211	498 (293–1519)	0.0005	0.0000	14%	10%	No
Bondalselva	9	0	209	1283 (418-Inf)	12	3	NC.	34# (26–47)	0.0043	0.0017	6%	0%	No
Ørstaelva	6	1	214	3678 (450-Inf)	17	0	210	400 (202–6501)	0.0003	−0.0013	0%	0%	No
GaulaSF	7	3	211	1193 (371-Inf)	19	2	205	439 (311–727)	0.0001	0.0008	17%	1%	No
Lærdalselva	8	1	193	Inf (−506-Inf)	13	2	200	333 (216–698)	0.0015	0.0010	15%	6%	No
Vosso	14	1	175	Inf (−304-Inf)	8	4	202	189 (138–294)	0.0179**	0.0213**	76%	67%	Yes
Loneelva	17	5	176	984 (348-Inf)	8	2	200	241 (172–390)	0.0120**	0.0116**	52%	29%	Yes
Opo	10	1	166	Inf# (−14-Inf)	58	1	184	68 (60–76)	0.0258**	0.0279**	100%	90%	Yes
Etne	25	1	209	752 (439–2405)	12	3	209	917 (507–4135)	0.0006	0.0000	5%	5%	No
Figgjo	9	1	204	Inf (−1638-Inf)	14	2	210	Inf (1070-Inf)	0.0048**	0.0058**	38%	4%	Yes
Numedalslågen	9	1	194	Inf (1194-Inf)	14	1	210	653 (383–2050)	0.0032*	0.0051*	29%	18%	No
Berbyelva	19	0	156	81 (67–101)	19	4	166	245 (194–327)	0.0053**	0.0071**	16%	7%	Yes

Within samples: LD = observed number of deviations from linkage disequilibrium (231 pair-wise tests per population, 211 tests for Opo) at α 0.05, HW = observed deviations from Hardy Weinberg Equilibrium (22 tests per population, 21 tests for Opo) at α 0.05, A_R_ = allelic richness computed using re-sample size of 25 (note Opo samples only computed with 21 loci therefore not directly comparable to other populations), *Ne* = effective population size as computed from LD method in LDNE [90] Inf = Infinity suggesting that the population is “relatively large” (i.e., >200) [93], # = harmonic mean sample size less than 30 and therefore estimated *Ne* not to be trusted. Between temporal samples: * = F_ST_ significant at α 0.05, ** = F_ST_ significant at α 0.001 (and following Bonferroni), NC = not computed, Exclusion from hist. = percentage of fish from the contemporary population that are excluded from the historical population profile in the program Geneclass at a cut off of α 0.001, temporal change ? = whether significant temporal genetic change is reported within rivers at α 0.001 based upon pair-wise F_ST_ for both sets of microsatellites.

When computed for all combinations of pairs of loci, within each population separately, LD was detected 309 (6.4%) and 35 (0.7%) times among the historical samples, 122 (6.6%) and 12 (0.6%) times in the intermediate samples, and 422 (8.7%) and 25 (0.5%) times in the contemporary samples at α 0.05, and α 0.001, respectively. Deviations were distributed evenly among the different combinations of pairs of loci, but unevenly distributed among the samples (Table 3). For example, in the historical samples, Vestre Jacobselv displayed 85 pair-wise LD combinations among loci (28% of all LD observed in the historical samples). Together, HWE and LD suggest some form of disturbance in the Vestre Jacobselv in the historical sample. Within the contemporary samples, three populations (Rokdalsvassdraget, Reisa and Opo) accounted for 44% of the pair-wise LD combinations observed.

All loci displayed statistically significant global F_ST_ estimates in the historical and contemporary data sets (Table S3). Samples corresponding to the historical data set identified three loci under possible directional selection (*MHC2, SsaF43, Ssa289*) and five under possible stabilizing selection (*SSsp2216, Ssa197, SsaD157, SsaD144, SSsp2201*), whereas the contemporary data set showed the same loci under possible directional selection but only two of the former ones under possible stabilizing selection (*SsaD157, SSsp2201*) (Text S1). Subsequently, analyses have been conducted on data sets comprised of the full (all 22 loci) and the neutral (14 loci only) markers.

### Temporal Genetic Variation within Populations

The number of alleles observed among populations, and between temporal samples within populations varied greatly (Table S3). Differences in sample size were accounted for by computing allelic richness A_R_. Looking specifically at temporal variation of A_R_ within populations, most showed a very slight increase with time, however, the populations Vosso, Opo and Loneelva increased by 18–27 (Table 3).

When considering data from the set of 22 loci, and the 14 neutral ones separately, statistically significant temporal genetic change, as measured by F_ST_, was detected in 6 of the 21 populations (Table 3). Populations displaying LD, or distinctly increased A_R_ in the contemporary samples, were all among those displaying temporal genetic changes. In three of the populations the F_ST_ estimates between historical and contemporary samples exceed 0.01 (*i.e.,* Opo, Vosso and Loneelva). The change in A_R_ from the historical to the contemporary samples was significantly higher (*P*  = 0.003; non-parametric Mann-Whitney test) in the six populations showing temporal genetic changes (mean increase per population  = 15.8), than in the six ones displaying the strongest temporal stability (mean increase per population = 2.6).

**Figure 2:**
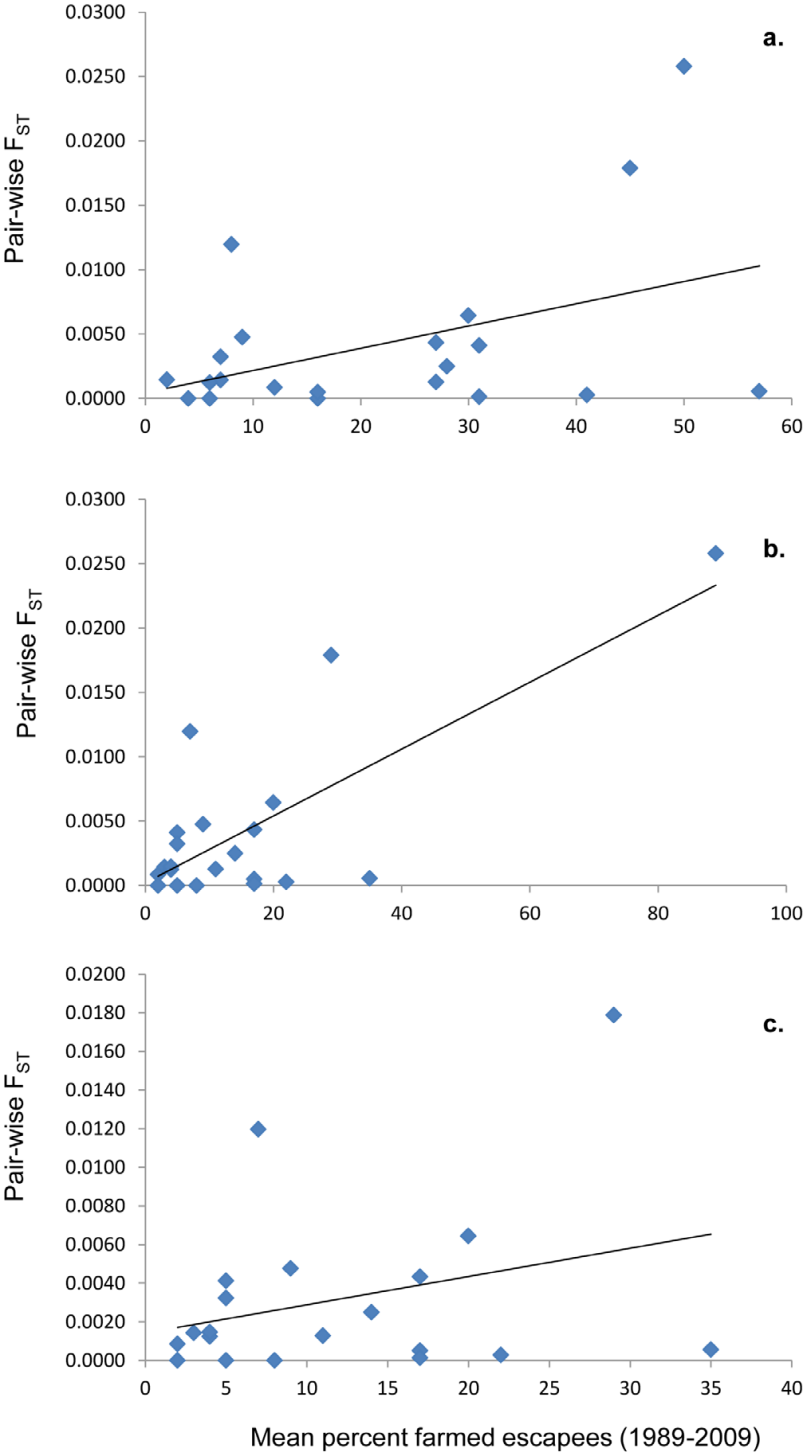
Relationship between average numbers of escapees observed in each population in the period 1989–2009, and the observed within-river temporal genetic changes as computed by pair-wise F_ST_ between the historical and contemporary sample. Graph a = relationship when using an un-weighted mean of the farmed escapees recorded in the autumn survey data (R^2^ = 0.18, *P = *0.052), graph b =  relationship when using a weighted mean based upon a mixture of summer sports fishing and autumn survey data (R^2^ = 0.56, *P<*0.0001) [52], and c = same as b with the population Opo excluded (R^2^ = 0.09, *P = *0.20).

No statistically significant correlation was observed between the frequency of farmed escapees observed in a given population in the period 1989–2009 based upon the un-weighted mean from the autumn spawning surveys (see Table 2), and pair-wise F_ST_ between the historical and contemporary samples for the same population (R^2^ = 0.18, *P = *0.052) (Fig. 2a). When using the weighted mean number of escapees reported in a combination of the summer sports-fishing catch and the autumn spawning counts for each population [52], the correlation with pair-wise F_ST_ was statistically strong (R^2^ = 0.56, *P<*0.0001) (Fig. 2b). However, when the river Opo was excluded (this river displayed by both the highest percentages of escapees and greatest temporal genetic change) the correlation was not significant (R^2^ = 0.09, *P  = *0.20) (Fig. 2c). The lack of a clear relationship between percentage of farmed fish (by either of the two estimations) and observed genetic changes is readily illustrated by the fact that two of the populations (e.g., Opo and Vosso) displayed high numbers of escapees on the spawning grounds and large temporal genetic changes, while other populations (e.g., Ørsta and Etne) also displayed high numbers of escapees but did not reveal genetic change with time. Furthermore, several other rivers had been exposed to >10% escapees in the period 1989–2009 without displaying statistically significant temporal genetic changes (Table 2, 3, Fig. 2).

**Figure 3:**
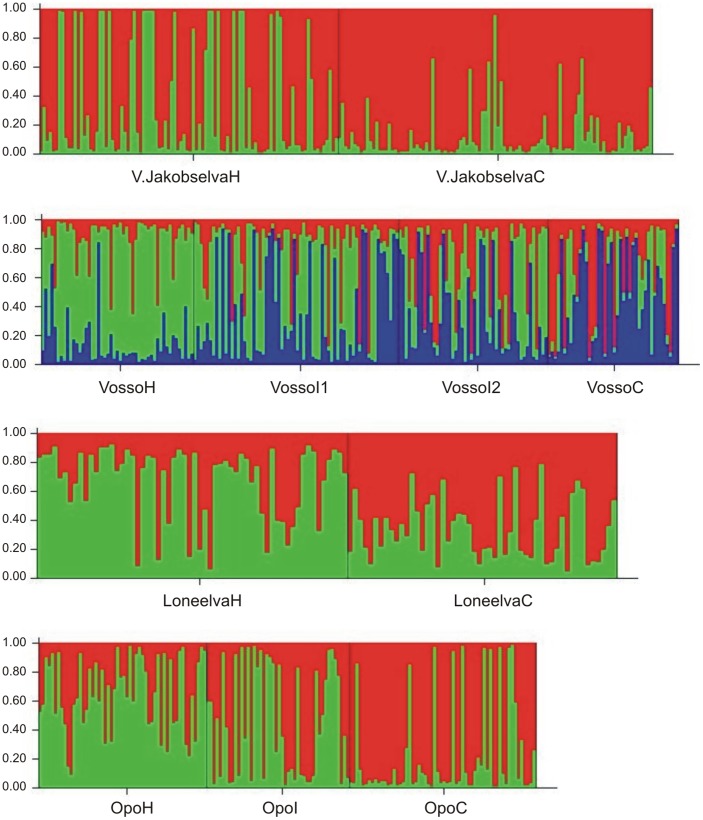
Bayesian clustering of historical (H), intermediate (I) and contemporary (C) samples representing the four rivers displaying the largest temporal genetic changes at 22 microsatellite loci. For the river Vosso, a total of four samples were available. Thus, the two intermediate samples for this river include a suffix I1 and I2 (linking to these specific samples to Table 1). These analyses were conducted on each river separately. Inferred ancestry was computed using STRUCTURE v. 2.3.3 [83], [84], under a model assuming admixture and correlated allele frequencies without using population information. Ten runs with a burn-in period consisting of 100000 replications and a run length of 1000000 Markov chain Monte Carlo (MCMC) iterations were performed for a number of clusters ranging from K 1 to 5. Then an ad hoc summary statistic ΔK [86] was used to calculate the number of clusters (K) that best fitted the data for each river separately. For full computation details and results for all populations using both 22 and 14 markers see Fig. S1 (supporting information).

Individual admixture analysis was also applied to evaluate within-population temporal stability, using historical, intermediate (when available) and the contemporary samples both for the total and neutral sets of microsatellites. The assessment of ▵K in single-population assignment analyses revealed that the most likely number of clusters ranged between two and three (Fig. 3; Fig. S1), although in one population, Berbyelva, this was ≥4 [86]. Admixture analysis supported the results of temporal change from the *F*-statistics. Thus, populations such as Opo, Vosso, Loneelva and Vestre Jakobselv, which showed temporal genetic changes in F_ST_, also showed evident signs of admixture (Fig. 3).

The percentage of fish from each contemporary sample that was excluded from its historical population sample when conducting genetic assignment ranged from 0–100% when using all 22 loci, and 0–90% when using the reduced set of neutral loci (Table 3). There was a strong correlation between percentage of fish that were excluded from their respective historical populations, and the pair-wise F_ST_ values (R^2^ = 0.86 *P*<0.0001). For example, the populations Opo, Vosso and Loneelva displayed the highest pair-wise F_ST_ values between historical and contemporary samples (0.028, 0.021, 0.012 respectively) in addition to the highest exclusion rates (100%, 76%, and 52% respectively). While other assignment methods implemented in the program Geneclass gave different absolute exclusion percentages, the above trend remained.

### Spatio-temporal Genetic Variation

**Figure 4:**
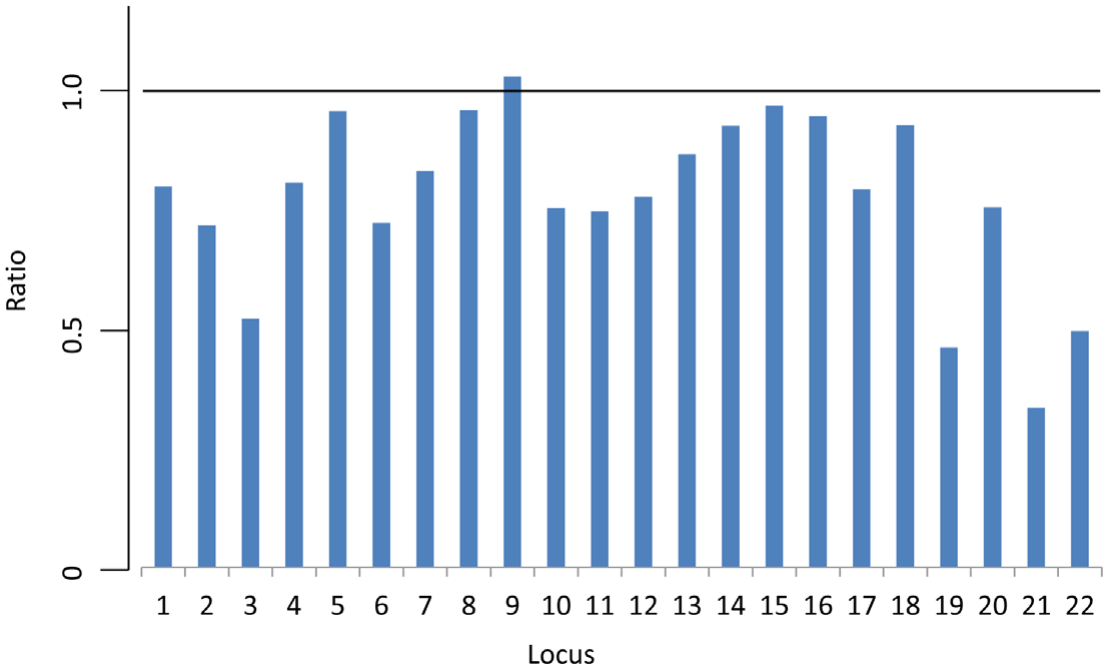
Ratio between global F_ST_ computed among the 21 contemporary samples divided by the global F_ST_ computed among the 21 historical samples for 22 microsatellite markers. Locus number is consequent with locus names and other locus-specific details available in Table S3.

Global F_ST_ among the 21 historical samples was significantly larger than among the 21 contemporary ones (Table 4). Significantly, the reduction in global F_ST_ with time was observed in 21 of the 22 loci (Fig. 4, Table S3).This trend was also reflected in the self-assignment analyses conducted in Geneclass which showed a drop from 61.6% of fish correctly assigned to their source populations in the historical data set, to 57.6% in the contemporary. Finally, the AMOVA analysis revealed that the amount of genetic variation observed among populations dropped from 4.1% in the historical data set to 2.9% in the contemporary one.

**Table 4:** Summary of global F_ST_ estimates, and, *P* values indicating whether the global F_ST_ estimates are significantly different between the historical and contemporary samples.

	COMPARISON F_ST_ BETWEEN GROUPS (Historical *vs*. contemporary)					
	TOTAL LOCI			NEUTRAL LOCI		
	F_ST_ histor.	F_ST_ contemp.	*P* value	F_ST_ history.	F_ST_ contemp.	*P* value
All 21 populations	0.038	0.030	0.008	0.038	0.028	0.001
20 populations (excluding Opo)	0.038	0.030	0.010	0.034	0.026	0.006
12 populations in restricted data set*	0.039	0.032	0.078	0.032	0.025	0.042
6 populations displaying temporal changes	0.058	0.039	0.006	0.057	0.032	0.001
6 populations displaying the strongest temporal stability	0.027	0.028	0.550	0.027	0.026	0.470

All global F_ST_ estimates were significant at α 0.001.

* These 12 populations were selected due to narrow the historical temporal data-set to the period 1986–1994, Opo was excluded due to the fact that it was only genotyped for 21 of the 22 loci.

The historical data set was drawn from a wider time-interval than the contemporary one (Table S1). Therefore, in order to test whether this was spuriously responsible for the drop in global F_ST_ between the two data sets, a reduced historical data set was established from 12 populations where samples were available from the interval 1986–1994. Likewise, a temporal reduction in global F_ST_ was still observed for the 12 populations (Table 4).

Looking specifically at the six populations displaying temporal genetic changes, global F_ST_ decreased from 0.058 among the historical samples, to 0.039 among the contemporary ones. In contrast, global F_ST_ estimated among the six populations that showed the highest level of within-river temporal stability did not display any change between the historical (0.026) and contemporary (0.027) data sets. Inspection of the pair-wise F_ST_ values among the six populations displaying within-population changes showed that all of them contributed to the distinct temporal decrease in global F_ST_ (Table 5, 6).

**Table 5:** Pair-wise genetic distance as computed by F_ST_ among the 6 populations displaying within-river temporal genetic changes. Computed for historical (bottom left) and contemporary samples (top right), and based upon the analysis of 22 loci.

	V. Jakobselva	Loneelva	Vosso	Opo	Figgjo	Berbyelva
**V. Jakobselva**		0.035	0.026	0.031	0.035	0.074
**Loneelva**	0.056		0.013	0.017	0.020	0.063
**Vosso**	0.067	0.048		0.008	0.014	0.051
**Opo**	0.061	0.038	0.033		0.015	0.051
**Figgjo**	0.055	0.047	0.037	0.039		0.042
**Berbyelva**	0.086	0.086	0.078	0.069	0.053	

Computed for historical (bottom left) and contemporary samples (top right), and based upon the analysis of 22 loci.

All F_ST_ values significant at α 0.001 with the exception of those in bold.

**Table 6:** Pair-wise genetic distance as computed by F_ST_ among the 6 populations displaying the greatest within-river temporal stability.

	Alta	Målselva	Eira	Ørstaelva	GaulaSF	Etne
**Alta**		0.020	0.056	0.054	0.046	0.051
**Målselva**	0.021		0.029	0.024	0.023	0.026
**Eira**	0.053	0.031		0.012	0.015	0.012
**Ørstaelva**	0.051	0.029	0.009		**0.003**	**0.002**
**GaulaSF**	0.049	0.027	0.009	0.007		0.004
**Etne**	0.053	0.035	0.012	0.006	0.005	

Computed for historical (bottom left) and contemporary samples (top right), and based upon the analysis of 22 loci.

All F_ST_ values significant at α 0.001 with the exception of those in bold.

Using data from all 22 markers, a significant relationship between geographic and genetic distance was observed for the total set of populations both in the historical (R^2^ = 0.365, *P<*0.0001) and in the contemporary samples (R^2^ = 0.377, *P<*0.0001). When looking specifically at the six populations not displaying temporal genetic change, a strong relationship was found in the historical (R^2^ = 0.758, *P = *0.0011), and contemporary data sets (R^2^ = 0.668, *P* = 0.0013). When examining the six populations displaying temporal genetic change, the relationship between genetic and geographic distance was not statistically significant in either the historical (R^2^ = 0.279, *P = *0.1013) nor the contemporary data sets (R^2^ = 0.221, *P* = 0.1411).

**Figure 5:**
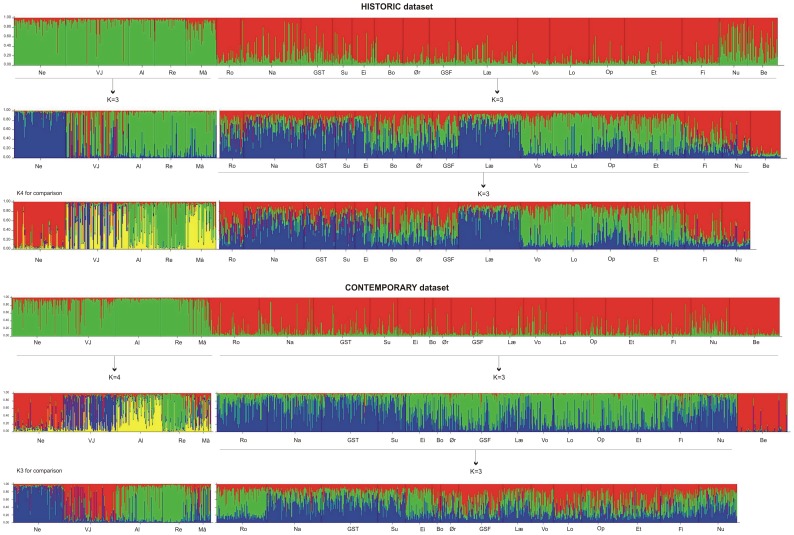
Hierarchical Bayesian clustering for the historical and contemporary data sets for 21 populations genotyped at 22 microsatellite loci. Inferred ancestry was computed using STRUCTURE v. 2.3.3 [83], [84], under a model assuming admixture and correlated allele frequencies without using population information. Ten runs with a burn-in period consisting of 100000 replications and a run length of 1000000 Markov chain Monte Carlo (MCMC) iterations were performed for a number of clusters ranging from K 1 to 5. Then, the ad hoc summary statistic ΔK [86] was used to calculate the number of clusters (K) that best fitted the data. Populations are ordered North to South, thus corresponding with Tables 1 and 2. Barplots for K3 and K4 are presented for comparison between the historical and contemporary data sets (see results section). For full computation details and results using both 22 and 14 markers see Fig. S2 (supporting information).

Admixture analyses conducted on the 21 populations provided the strongest support for K = 2, both when considering the probability of the data [P(D)] and the ad hoc statistic ΔK, for historical and contemporary samples when using the 22 loci (Fig. 5) and the 14 neutral loci (Fig. S2). In both cases, the five northernmost populations formed a very distinct separate cluster. Following a hierarchical approach, we split the data set into the corresponding five and sixteen populations respectively and conducted the assignment analyses separately. Looking at the full set of markers, the five northernmost populations yielded K3 in the historic dataset and K4 in the contemporary one. Visual inspection of either K3 or K4 for the northern populations revealed increased admixture in several of the rivers over time. This was most apparent for the rivers Vestre Jakobselv, and interestingly, Målselva, the latter of which did not display temporal genetic change as computed by *F*_ST_, nor by single-river admixture analysis (Fig. S1). Turning to the remaining sixteen populations, both the historical and contemporary data sets revealed K = 3 as the most likely number of clusters. The southernmost population, Berbyelva was the most distinct (especially in the contemporary data set), and therefore, admixture analyses were also computed with this population excluded. Changes in genetic structure between the historical and contemporary data sets across these sixteen populations were subtle, and not as distinct as for changes within populations (Fig. 3; Fig. S1).

**Table 7:** *P*-values testing whether the observed pair-wise F_ST_ between each population’s historical and contemporary sample was significantly larger than the F_ST_ between each population’s observed historical sample and 1000 computer simulated contemporary samples.

*Ne*	Population					
	V. Jakobselv	Vosso	Loneelva	Opo	Figgjo	Berbyelva
**25**	0.99	0.99	0.85	1.0	1.0	0.97
**50**	0.3	0.4	0.03	0.57	1.0	0.2
**75**	0.04	0.01	0.02	0.01	1.0	0.01
**100**	<0.001	<0.001	<0.001	<0.001	0.95	<0.001
**200**	<0.001	<0.001	<0.001	<0.001	0.03	<0.001
**300**	<0.001	<0.001	<0.001	<0.001	<0.001	<0.001
**500**	<0.001	<0.001	<0.001	<0.001	<0.001	<0.001

**Figure 6:**
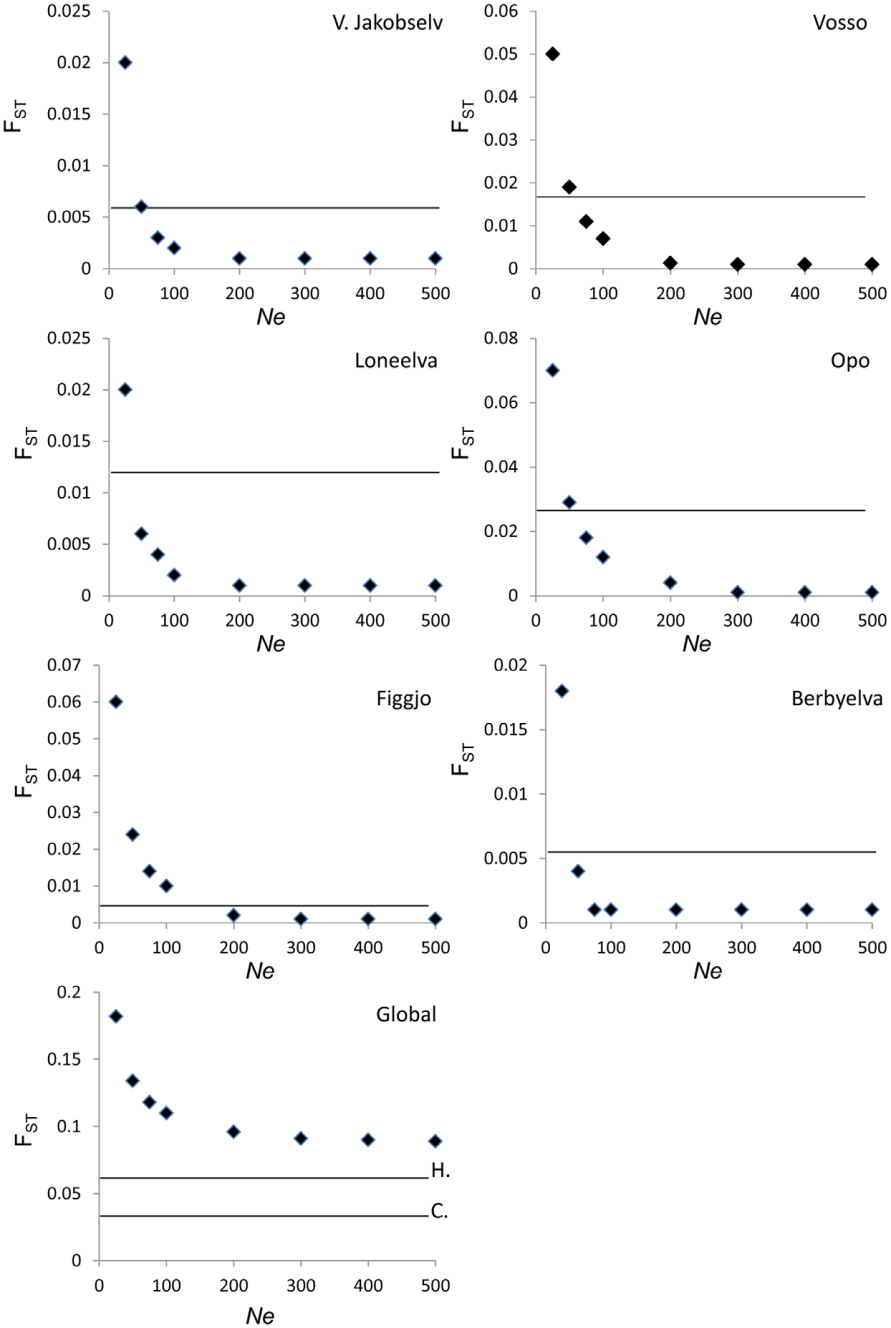
Simulations of genetic drift induced changes between the observed historical genetic profile and computed contemporary populations for each of the six populations displaying temporal genetic change. Black diamonds represent the mean F_ST_ between the historical population and the computed contemporary population based upon 1000 simulations of genetic drift with *Ne* set to 25, 50, 75, 100, 200, 300, 400 and 500. Horizontal black line for each plot represents the observed pair-wise F_ST_ between the historical and contemporary population (i.e., the values given in Table 2). “Global” plot represents the global F_ST_ computed among these six populations based upon the above mentioned simulations, while the horizontal black bar H = historical global F_ST_ observed among these populations, and C  =  contemporary global F_ST_ observed among these populations (i.e., the values given in Table 3). Statistical significance levels for these comparisons are presented in Table 5.

Simulations were based upon genetic drift at different *Ne*. Plots of observed and simulated F_ST_ values are presented in Fig. 6.

In order to investigate whether the inclusion of farmed salmon would improve the power to detect temporal genetic changes in population genetic structure (either within or among populations), samples from nine genetically distinct farm sources were included in the admixture analyses. Runs were conducted for K = 12 and K = 13 as the analyses included salmon from 9 distinct farm samples, and, that K for the northern and southern clusters had already been estimated at 3 or 4. Both sets of analyses were conducted with and without a prior for the farm samples (which made no difference to the result). As expected, samples from the farms were confirmed to be highly distinct to each other, whereas wild populations were strongly admixed in both the historical and contemporary samples (Fig. S3). Thus, inclusion of farmed fish did not reveal additional temporal genetic changes not already detected.

### Effective Population Size and Simulations of Genetic Drift

In most of the historical and contemporary samples representing each population, the computed effective population size (*Ne*) was larger than 200 (Table 3). Confidence intervals associated with these estimates were large, often reaching infinity in the upper bound (Table 3, Table S4). Several of the samples also showed negative values, both in the upper and lower bound. Negative values occur when the variance observed can be ascribed entirely to sampling error alone, and suggests that these samples displayed relatively high *Ne* (i.e., >200) [93].

Simulations of genetic drift were conducted for the six populations identified as displaying statistically significant temporal genetic changes. These simulations were conducted in order to evaluate the possibility that genetic drift could have caused the observed changes given the number of generations that have occurred between the historical and contemporary samples.

Unsurprisingly, the mean pair-wise F_ST_ between the historical sample and the simulated contemporary population was strongly influence by *Ne* (Fig. 6); small *Ne* leading to large F_ST_. For five of the six populations, a value of *Ne* of 100 was sufficient to exclude genetic drift as the primary driver of the observed temporal genetic changes (*P*<0.001). In these cases the pair-wise F_ST_ that was observed between the historical and contemporary sample was greater than the pair-wise F_ST_ between the historical sample and the simulated population in all the replicates (i.e., *P*<0.001 for 1000 replicates). In the river Figgjo, a value of *Ne* of 300 or more would be required to achieve the same level of significance (*P*<0.001). Comparing these genetic drift simulations with the computed *Ne* values (Table 3) revealed that genetic drift can be confidently excluded as the driver of the observed temporal genetic changes in the rivers Vosso, Loneelva and Figgjo. This is due to the fact that their *Ne* values ranged between several hundred and infinity in both the historical and contemporary samples (Table 3). For the rivers V. Jakobselv, Opo and Berbyelva, either the historical or contemporary sample displayed a *Ne* lower than 100 (79, 68 and 81 respectively). This is at the level of *Ne* where the potential for genetic drift to contribute to temporal genetic changes on the time-scale studied can be excluded at modest levels of statistical significance (*P*  = 0.04, 0.01, and 0.01 for V. Jacokbselv, Opo and Berbyelva respectively for *Ne*  = 75) (Fig. 6, Table 7). Nevertheless, all of these three populations displayed *Ne* values >150 in one of the samples.

Strong genetic drift in small populations is not only expected to lead to within-population temporal instability, it is expected to simultaneously lead to increased inter-population differentiation (on average) when it is stronger than the influence of gene-flow [88], [89]. The genetic drift based simulations reported above were also used to re-compute the global F_ST_ value between the six populations displaying statistically significant temporal genetic changes after having simulated genetic drift independently within each (Fig. 6). The “global” plot illustrates that as *Ne* decreases, and genetic drift becomes more pronounced within each population, the level of inter-population genetic differentiation increases rapidly. This is in stark contrast to the large and statistically significant drop actually observed in the global F_ST_ among these six populations with time (Table 5).

## Discussion

This study represents one of the largest temporal analyses of population genetic structure conducted thus far. Samples covering an entire country, and spanning up to four decades, have permitted the identification of genetic changes occurring both within and among 21 populations, through time. Two main conclusions can be drawn from these analyses. First, despite the fact that farmed escapees have been recorded on the spawning grounds for all of the populations studied, outnumbering wild conspecifics in some years in some of the populations, only weak to moderate changes in among-population genetic structure have been observed in the time-period studied, and in most rivers, statistically significant temporal genetic changes were not observed. This demonstrates that generally, farmed escaped salmon have had poor to moderate success in the wild. Second, not all populations were equally resilient. Genetic changes were observed in six of the populations (29% of those studied), and in four of them, the changes were highly significant. For example, 100%, 76% and 52% of the fish comprising the contemporary samples for Opo, Vosso and Loneelva were excluded from their respective historical baseline samples at *P* = 0.001 and when using data from all 22 loci. At the same time, genetic drift was excluded as the primary contributing factor. These changes have occurred during 15–30 years, equivalent to approximately 3–6 generations in native populations. Thus, these data demonstrate that farmed Atlantic salmon have successfully introgressed and caused genetic changes in some wild Norwegian populations.

A weak to moderate but statistically significant reduction in population genetic structure was observed among the 21 populations with time. This is consistent with an increase in gene flow, and has been previously reported in response to extensive supplementation and translocations of brown trout in Denmark [46], among stocks of pearl oyster (*Pinctada margaritifera cumingii*) throughout French Polynesia [94], and among brook charr (*Salvelinus fontinalis*) populations in Canadian lakes [95]. Importantly, a reduction in population genetic structure is a predicted response to widespread gene flow from farmed escapees, based upon simulations conducted with genetic data in Norway [45], [47]. Nevertheless, although a decrease in population heterogeneity was observed with time, significant population genetic structure was still observed in the contemporary data set. Both the historical and contemporary datasets displayed a clear pattern of isolation by distance which is characteristic for Atlantic salmon [15], [16]. In 15 of the 21 populations, temporal genetic changes were not detected despite the fact that all of them had experienced farmed escapees on the spawning grounds, and in some years, escapees had outnumbered wild spawners (Table 2). While it is possible that the set of markers implemented here may have failed to detect low-levels of introgression in some populations (see discussion below), it is concluded that the gene flow from farmed escapees into native populations throughout Norway, has been less than the numbers of escapees observed on the spawning grounds. We suggest that this is primarily due to the fact that farmed escapees display reduced spawning success [96]–[98], in addition to the fact that their offspring display lower survival in the wild when compared with native conspecifics [96], [99], [100].

Not all of the populations studied were equally resilient. Statistically significant temporal genetic changes were observed in six populations, and for some of these, the changes were very distinct and highly significant. For example, 100%, 75% and 52% of the contemporary samples from Opo, Vosso and Loneelva were excluded from their respective historical profiles. When focusing on the six populations displaying temporal changes, global F_ST_ nearly halved between the historical and contemporary data sets. From population genetics theory [88], classical experimental studies [89], and the simulations conducted within this study, genetic drift is expected to lead to greater differentiation among populations. This has been documented for example in the Spanish imperial eagle (*Aquila adalberti*) [101] and forest jaguars (*Panther onca*) [102] in response to habitat fragmentation, and among Atlantic salmon populations that have experienced significant population declines at the southernmost part of their natural distribution [103]. In addition, none of the six populations displaying temporal genetic changes had very low *Ne* estimates, and based upon simulations, genetic drift was conclusively excluded as the primary driver of the observed temporal genetic changes within most of these rivers. Furthermore, genetic drift was demonstrated to be incompatible with the observed drop in differentiation among these populations with time, and not least, cannot explain the increase in the number of alleles observed in all of these populations. Therefore, in consideration of the genetic data and simulations presented, characteristics of these populations, the high numbers of escapees observed on the spawning grounds (Table 2), and the fact that successful spawning of farmed escaped salmon has been documented in several Norwegian rivers in the time period studied [104], [105], it is concluded that genetic introgression of farmed escaped salmon represents the primary cause of the observed temporal genetic changes. Specifically in the case of the river Vosso, extensive spawning of farmed females has been documented by size and pigment measurements conducted on eggs deposited in the river, leading to the conclusion that the population in this river had been replaced by farmed escapees in the 1990’s [105]. The results of that field experiment are highly consistent with both the timing and magnitude of genetic changes observed in the river Vosso in the present study. Nevertheless, it is worthy of note that the populations in Berbyelva and Figgjo both displayed relatively small temporal genetic changes. For these two populations, the influence of non-biological factors, for example sampling bias in the historical or contemporary samples, or unidentified natural or anthropogenic disturbances, may have had a proportionately high contribution to the observed changes.

No clear relationship between the reported frequency of farmed fish in each population, and the degree of within river genetic changes were revealed in this study. This was true when using both the unweighted mean percent of farmed fish observed in the autumn survey, and the weighted mean combining data from summer sports-fishing catches and autumn surveys [52] (in combination with removing the single river sample Opo which was solely responsible for the statistically significant relationship) (Fig. 2a, b, c). There are many potential explanations for this result. Firstly, it is important to consider the fact that the numbers of rivers investigated is only 21, limiting the ability to test for such a relationship in a statistically robust manner. Furthermore, and importantly, the data relating to the frequency of farmed fish in these populations (either the summer sports-fishing data or the autumn surveys) has limitations, such as missing counts in some years (Table 2), and the fact that the maturity status of these escapees is not often recorded. Nevertheless, the question still remains; why did some populations (e.g., Opo and Vosso) experiencing large numbers of domesticated escapees display very large temporal genetic changes, while other populations (e.g., Ørsta and Etne), also displaying high percentages of escapees, not reveal detectable temporal genetic changes? From both ecological and conservation viewpoints, these are vital questions in order to understand the evolutionary processes underlying the potential for natural populations to persist in the face of migration and potential gene flow from non-native sources. We suggest that there are both ecological and technical reasons for this. First we address the ecological reasons.

Farmed salmon are competitively inferior to wild salmon in spawning [96]–[98], and their relative spawning success is density-dependant [106]. Density-dependant spawning success has also been observed for hatchery reared salmon [107]. Together, these studies suggest that farmed escaped salmon will have a higher probability of introgression in native populations with low adult densities, than in populations with high adult densities. Once introgression has occurred, it is likely that the relative survival of the domesticated offspring and admixed individuals will be higher in rivers displaying low juvenile density and accordingly low intra-specific competition. This is because the offspring of domesticated and non-native conspecifics tend to display lower survival in the wild when compared to native fish [96], [99], [100]. This is consistent with the fact that successful introgression of hatchery reared brown trout in native Danish populations has been partially explained by low wild fish population density [46], and with a recent study that concluded that wild population density is the most important factor affecting the competitive balance between hatchery-reared and wild fish [108]. Furthermore, the two populations (Opo and Vosso) displaying the greatest genetic changes in the present study, have both experienced low numbers of adult spawners in the period where high numbers of escapees were reported. In contrast, two other populations (e.g., Ørsta and Etne) displaying relatively high numbers of wild adult spawners in the population, did not display temporal genetic changes, despite high numbers of escapees.

For several technical reasons, it is possible that the estimated level of within-population temporal genetic changes, as estimated by the 22 microsatellites implemented here, is lower than the *true* level of genetic introgression by farmed escapees. As detailed in the Materials and Methods, gene flow from farmed fish into wild populations may be concealed and thus underestimated [45]. Several of the populations studied here displayed close to significant temporal genetic changes in F_ST_, relatively high exclusion rates from the historical population, and, some evidence of linkage disequilibrium (Table 3). Furthermore, the ability to detect statistically significant temporal genetic changes is influenced by the ratio between sample and effective population size (S/*Ne*) [109]. Given that both factors varied among the samples and populations in this study (i.e., the contemporary sample for Bondalselva, which represented the smallest sample, was only N = 16), this may have limited the ability to detect temporal changes in some of the populations. It is possible however, that analysis of genetic markers putatively under domestication selection [44] may provide the ability to quantify introgression of escapees in rivers where this has occurred at a low level.

The effective population size (*Ne*) represents an important parameter in conservation genetics as it provides information about the potential for genetic drift, inbreeding and natural selection to act upon populations. A range of methods for computing *Ne* are available, and may be broadly split into temporal [109]–[112] and one-sample [90], [113]–[115] based approaches. Here, we applied a one-sample based method [90] that utilizes a bias correction [116]. This provided us with the ability to compute *Ne* for both the historical and contemporary samples separately, in order to estimate whether these were small populations likely to be under the influence of genetic drift. All methods of computing *Ne* include underlying assumptions that are rarely fulfilled in the populations in which they are implemented. For example, linkage disequilibrium, which is the primary parameter used to estimate *Ne* in single-sample methods, can be caused by several factors not related to *Ne*, such as immigration and overlapping generations. Both of these two underlying assumptions were violated by the populations in the present study, although the LD method implemented by [90] has been demonstrated to be robust to equilibrium migration [117]. Thus, while the *Ne* estimations presented here should be treated with some caution, they nevertheless provide indications regarding each population’s effective size, and thus potential for genetic drift.

The genetic changes observed here occurred over a period of 15–30 years, which is equivalent to approximately 3–6 generations for these wild populations. This time-scale is consistent with predictions from models of gene flow based upon experimental data in which it has been suggested that under high intrusion scenarios, it will be difficult to obtain broodstock from the original population after just a few generations [118]. This correlates strongly with the results of our genetic assignment tests, where over half of the contemporary populations for Opo, Vosso and Lonelva could be excluded from their historical population profiles at *P* = 0.001 (Table 3). Given that farmed salmon continue to escape into the natural environment, it is likely that the number of populations where introgression is observed, and the magnitude of introgression within each population, will increase with time. Several of the salmonid species in the Pacific are monitored, and in some circumstances, actively managed using genetic based methods [119]. Furthermore, there are a range of advantages in using genetic methods to monitoring populations for conservation and management [120]. Here, it is suggested that if farmed salmon continue to escape into the wild, a monitoring program to assess genetic stability in native salmon populations will be necessary in order to produce science-based management strategies in the future.

Salmonid fish populations are often regarded as locally adapted to their native environments [1]–[3], and supplementation with hatchery produced or non-native conspecifics is potentially negative to wild populations [121]. Farmed salmon have been selected for a range of economically important traits for approximately ten generations, and as a result, they display genetic differences to wild salmon. For example, farmed salmon grow significantly faster [122], transcribe genes differently [123], exhibit reduced anti-predator responses [124], and display lower fitness in the natural environment [96], [99], [100]. Nevertheless, analysis of neutral, or nearly neutral genetic markers as has been conducted here, can only describe changes in population genetic structure due to gene flow. While this represents a necessary step towards understanding the level of genetic-impact that farmed escaped fish may cause in native populations, such data cannot directly infer biological consequences in recipient wild populations. Ultimately, a major question will be how allele frequencies in genes causatively linked to adaptive traits have changed in these populations.

## Supporting Information

Figure S1 **Bayesian clustering of historical (H), intermediate (I) and contemporary (C) samples for 21 Atlantic salmon rivers separately.**
(DOC)

Figure S2 **Hierarchical Bayesian clustering of the 21 rivers in the historical and contemporary data sets.**
(DOC)

Figure S3 **Bayesian clustering of the 21 rivers in the historical and contemporary data sets when combined together with data from 9 distinct farm sources.**
(DOC)

Table S1 **Years in which samples were taken for the historical, intermediate, and contemporary data sets.**
(XLS)

Table S2 **Allele frequencies observed in the historical and contemporary data sets for 22 microsatellite markers.**
(XLS)

Table S3 **Locus by sample summary statistics for the historical, intermediate and contemporary samples collected from 21 Norwegian rivers.**
(XLS)

Table S4 **Effective population size for samples in the historical and contemporary data sets as computed by the LD method as implemented in LDNE** [**90**] **.**
(XLS)

Text S1 **Description of methods and results for identification of markers putatively under selection.**
(DOC)
