# Supplementary material for: Three Decades of Farmed Escapees in the Wild: A Spatio-Temporal Analysis of Atlantic Salmon Population Genetic Structure throughout Norway
Source: PLoS One. 2012 Aug 15;7(8):e43129. doi: 10.1371/journal.pone.0043129 (PMC3419752; doi:10.1371/journal.pone.0043129)
Supplement: Figure S1 — Bayesian clustering of historical (H), intermediate (I) and contemporary (C) samples for 21 Atlantic salmon rivers separately. (DOC) [file pone.0043129.s001.doc]

**Three decades of farmed escapees in the wild: a spatio-temporal analysis of Atlantic salmon population genetic structure throughout Norway**

Kevin A. Glover^1*^, María Quintela^2^, Vidar Wennevik^1^, François Besnier^1^, Anne G. E. Sørvik^1^, Øystein Skaala^1^

**Fig. S1. Supporting information.**

**Admixture analysis of 21 Atlantic salmon populations distributed throughout Norway.**

**Notes to figures.**

Sample size (N) and time span for historic (H), intermediate (I) when applies, and contemporary sampling (C), ordered in the barplot with increasing numbers. Inferred ancestry of individuals was calculated with STRUCTURE v.2.3.3 for a data set of 22 microsatellite and 14 microsatellite loci under a model assuming admixture and correlated allele frequencies without using population information. Ten runs with a burn-in period consisting of 100000 replications and a run length of 1000000 Markov chain Monte Carlo (MCMC) iterations were performed for a number of clusters ranging from K 1 to 5. Then, we applied the Evanno’s ad hoc summary statistic ΔK to calculate the number of clusters (K) that best fitted the data.

| **River** | **Sample** | **N** | **Time span** | **STRUCTURE barplot TOTAL MARKERS** | **STRUCTURE barplot NEUTRAL MARKERS** |
| --- | --- | --- | --- | --- | --- |
| Neiden | NeidenH | 79 | 1979-1982 | 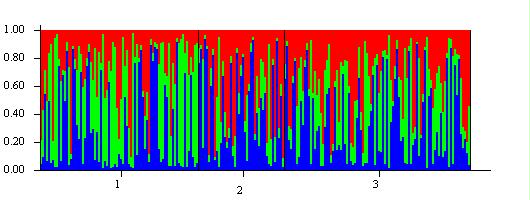 | 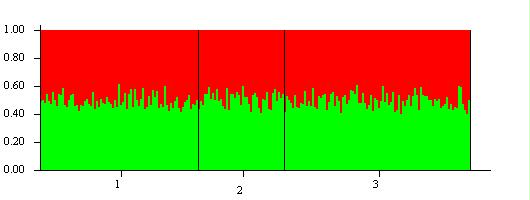 |
|  | NeidenI | 43 | 1989-1990-1991-1992-1993 |  |  |
|  | NeidenC | 93 | 2009 |  |  |
| Vestre Jakobselva | V.JakobsH | 96 | 1989-1990-1991 | 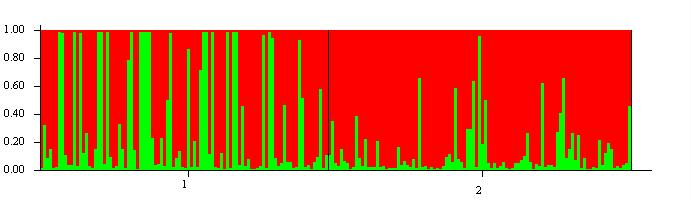 | 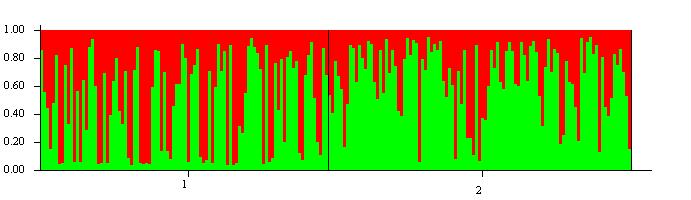 |
|  | V.JakobsC | 101 | 2007-2008 |  |  |

| **River** | **Sample** | **N** | **Time span** | **STRUCTURE barplot TOTAL MARKERS** | **STRUCTURE barplot NEUTRAL MARKERS** |
| --- | --- | --- | --- | --- | --- |
| Alta | AltaH | 39 | 1988-1989-1990 | 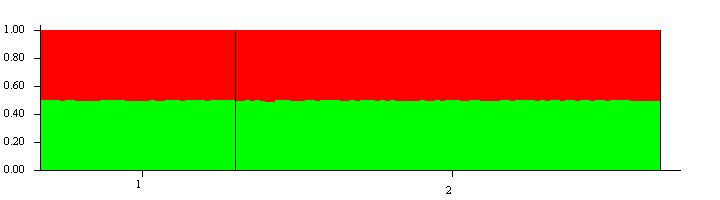 | 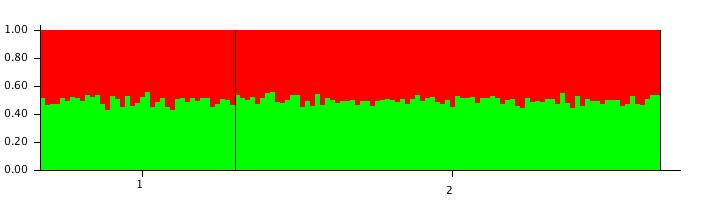 |
|  | AltaC | 85 | 2005-2007 |  |  |
| Reisa | ReisaH | 48 | 1986-1987-1988-1989-1990-1991 | 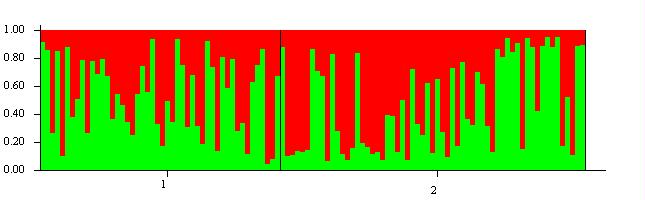 | 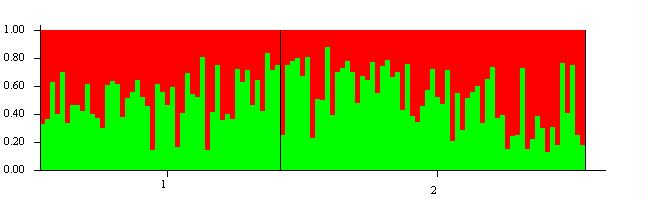 |
|  | ReisaC | 61 | 2006 |  |  |
| Målselva | MålselvH | 47 | 1986-1987-1988 | 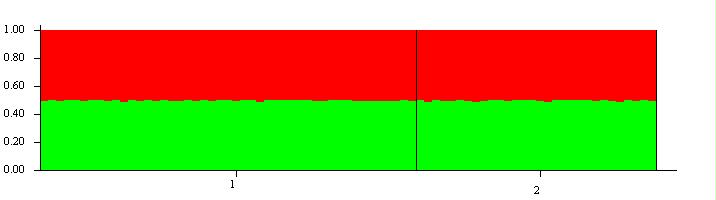 | 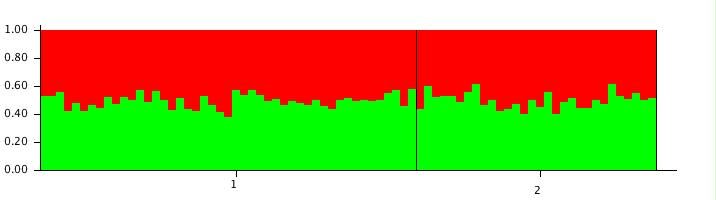 |
|  | MålselvC | 30 | 2008 |  |  |
| Roksdalsvassdragget | RoksdalsH | 37 | 1987-1988-1989-1990-1991-1992-1993 | 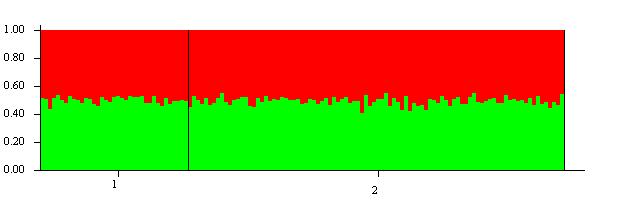 | 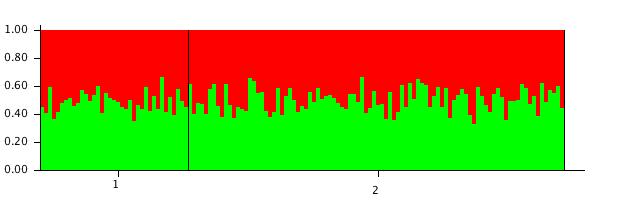 |
|  | RoksdalsC | 94 | 2008 |  |  |
| Namsen | NamsenH | 92 | 1977 | 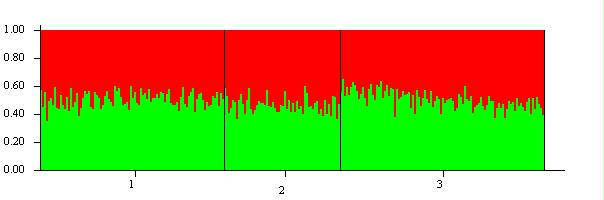 | 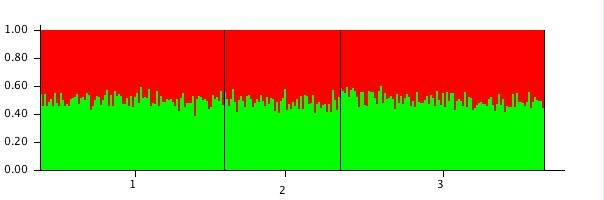 |
|  | NamsenI | 58 | 2000 |  |  |
|  | NamsenC | 102 | 2008 |  |  |

| **River** | **Sample** | **N** | **Time span** | **STRUCTURE barplot TOTAL MARKERS** | **STRUCTURE barplot NEUTRAL MARKERS** |
| --- | --- | --- | --- | --- | --- |
| Gaula ST | Gaula STH | 48 | 1986-1989-1990-1991-1992-1993-1994 | 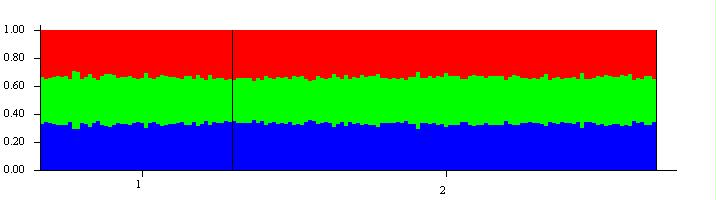 | 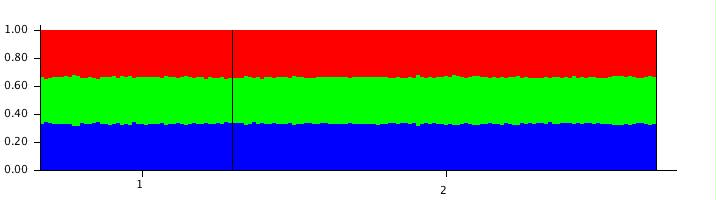 |
|  | Gaula STC | 106 | 2006-2007-2008 |  |  |
| Surna | SurnaH | 30 | 1986-1987-1988-1989 | 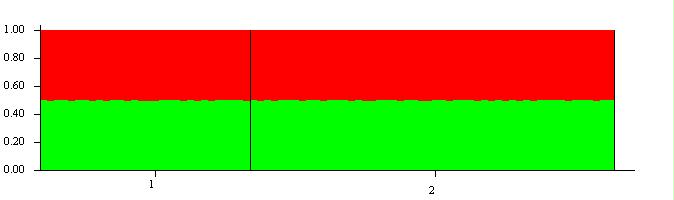 | 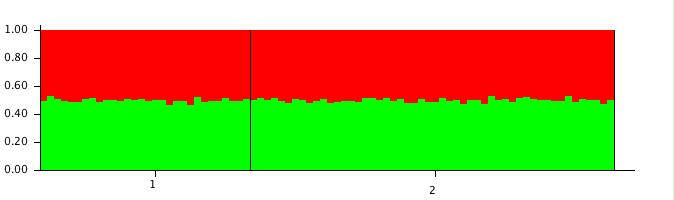 |
|  | SurnaC | 52 | 2005-2006-2007-2008 |  |  |
| Eira | EiraH | 34 | 1986-1987-1988-1992-1994 | 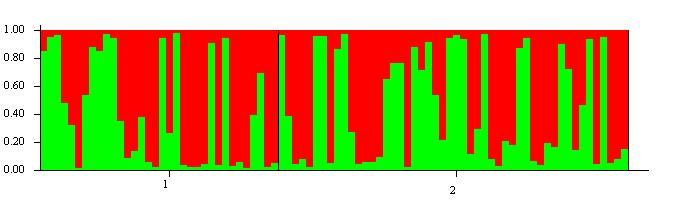 | 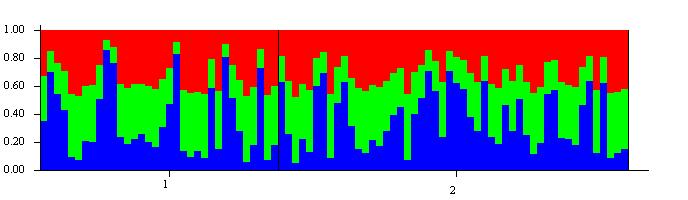 |
|  | EiraC | 50 | 2005-2006-2007-2008 |  |  |
| Bondalselva | BondalsH | 44 | 1986-1987-1988 | 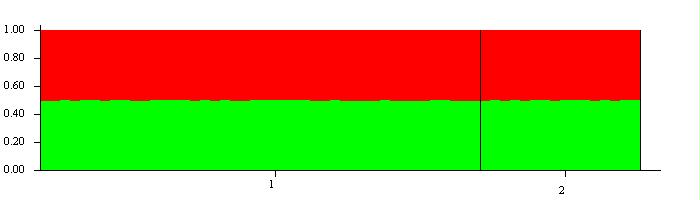 | 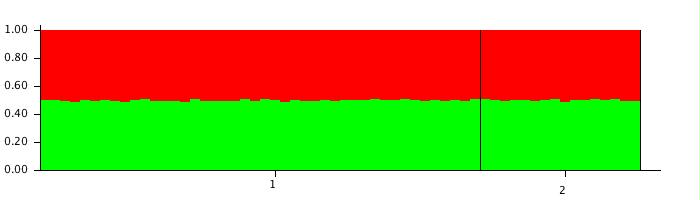 |
|  | BondalsC | 16 | 2007 |  |  |
| Ørstaelva | ØrstaH | 40 | 1986-1987-1988-1989 | 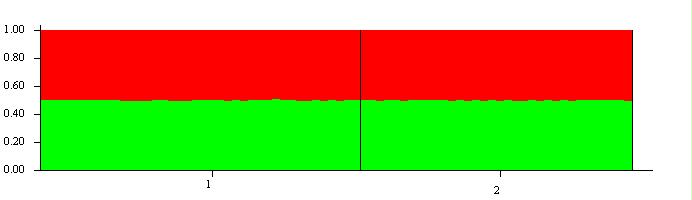 | 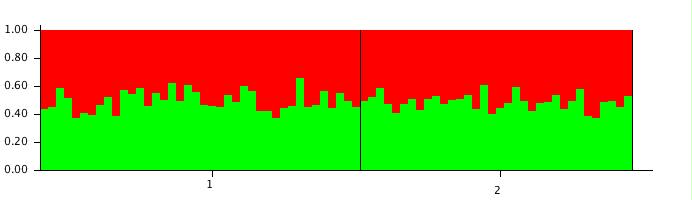 |
|  | ØrstaC | 34 | 2006-2008 |  |  |

| **River** | **Sample** | **N** | **Time span** | **STRUCTURE barplot TOTAL MARKERS** | **STRUCTURE barplot NEUTRAL MARKERS** |
| --- | --- | --- | --- | --- | --- |
| Gaula SF | Gaula SFH | 40 | 1987-1988-1989-1990-1991-1992-1993 | 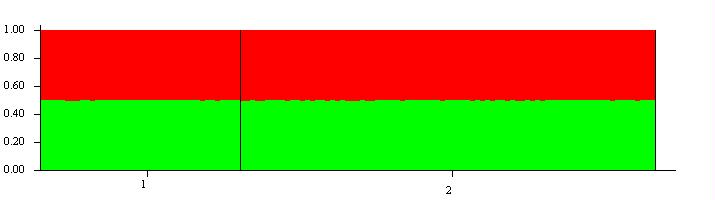 | 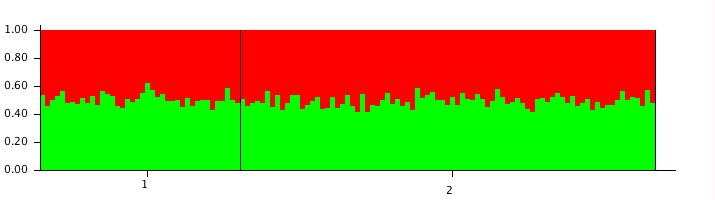 |
|  | Gaula SFC | 83 | 2006-2008 |  |  |
| Lærdalselva | LærdalsH | 95 | 1973 | 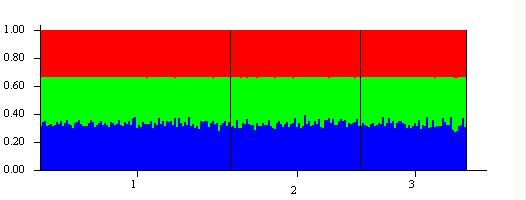 | 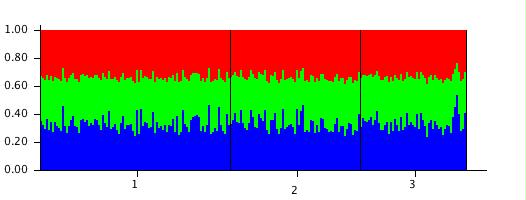 |
|  | LærdalsI | 65 | 1996-1997 |  |  |
|  | LærdalsC | 53 | 2005-2006-2007-2008 |  |  |
| Vosso | VossoH | 49 | 1980 | 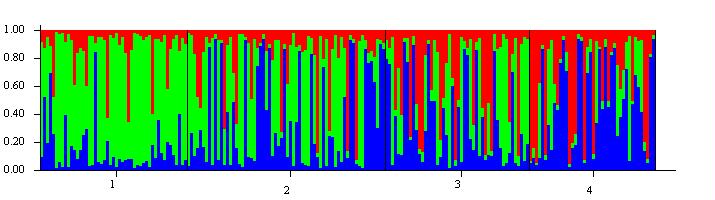 | 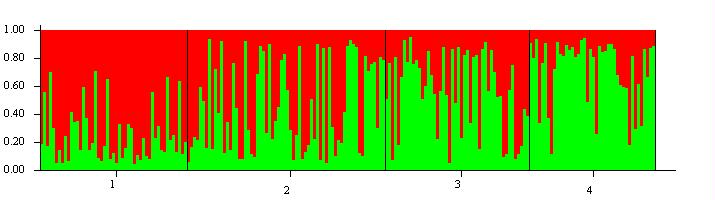 |
|  | VossoI1 | 66 | 1993-1994-1995-1995-1996 |  |  |
|  | VossoI2 | 48 | 2007-2008 |  |  |
|  | VossoC | 42 | 2008 |  |  |
| Loneelva | LoneH | 60 | 1986-1987-1988-1989-1993 | 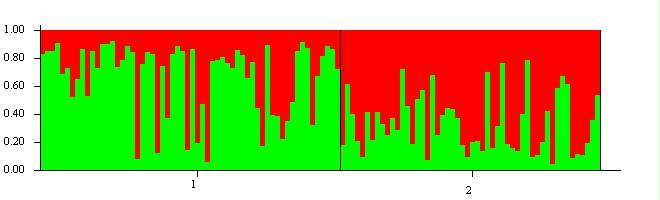 | 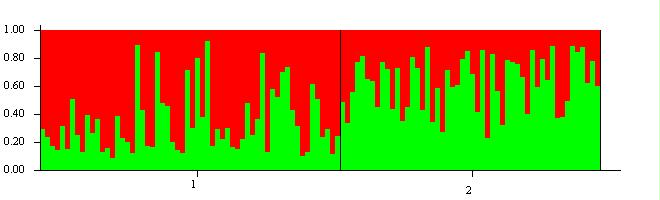 |
|  | LoneC | 52 | 2001-2005-2006-2007 |  |  |

| **River** | **Sample** | **N** | **Time span** | | **STRUCTURE barplot TOTAL MARKERS** | | **STRUCTURE barplot NEUTRAL MARKERS** | |
| --- | --- | --- | --- | --- | --- | --- | --- | --- |
| Opo | OpoH | 54 | | 1971-1973 | | 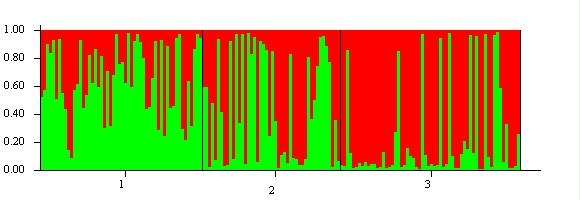 | | 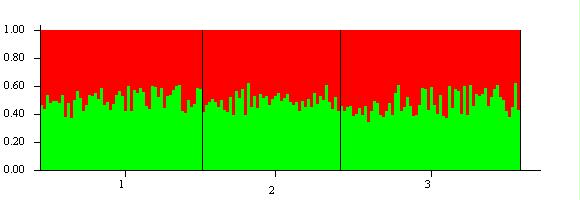 |
|  | OpoI | 46 | | 2000 | |  |  |  |
|  | OpoC | 60 | | 2010 | |  |  |  |
| Etne | EtneH | 88 | 1983 | | 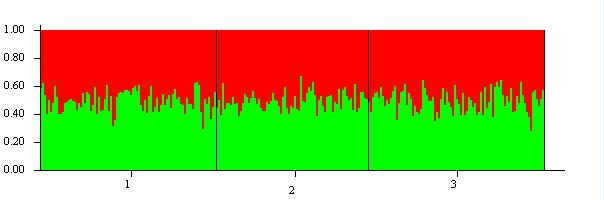 | | 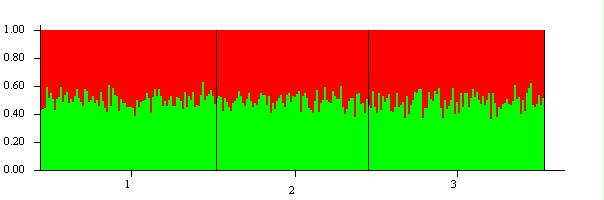 | |
|  | EnteI | 76 | 1997-1998 | |  |  |  |  |
|  | EtneC | 99 | 2006-2007-2008 | |  |  |  |  |
| Figgjo | FiggjoH | 57 | 1972-1973-1974-1975 | | 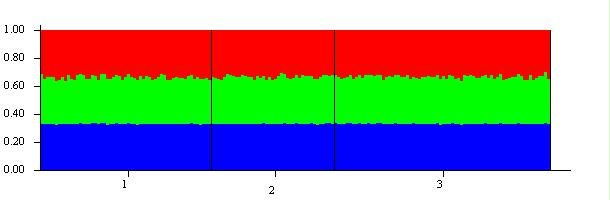 | | 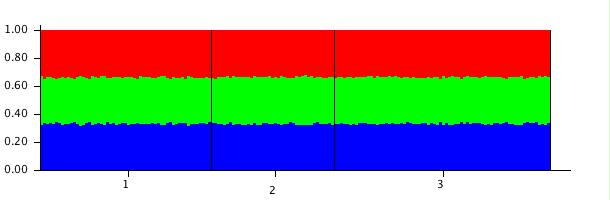 | |
|  | FiggjoI | 41 | 1987-1989-1990 | |  |  |  |  |
|  | FiggjoC | 72 | 2006 | |  |  |  |  |
| Numedalslågen | NumedalsH | 43 | 1989-1990-1991-1992-1993 | | **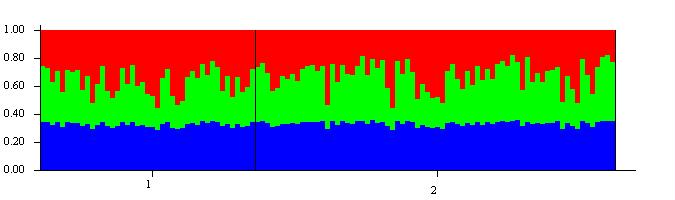** | | 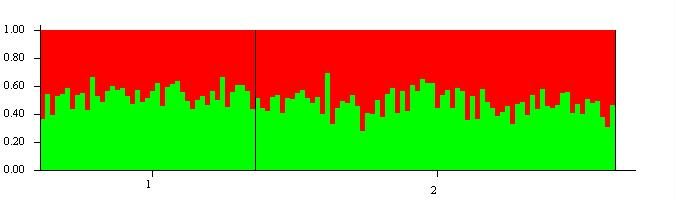 | |
|  | NumedalsC | 72 | 2007-2008 | |  |  |  |  |

| **River** | **Sample** | **N** | **Time span** | **STRUCTURE barplot TOTAL MARKERS** | **STRUCTURE barplot NEUTRAL MARKERS** |
| --- | --- | --- | --- | --- | --- |
| Berbyelva | BerbyelvaH | 46 | 1988-1989-1990-1991-1992-1993 | **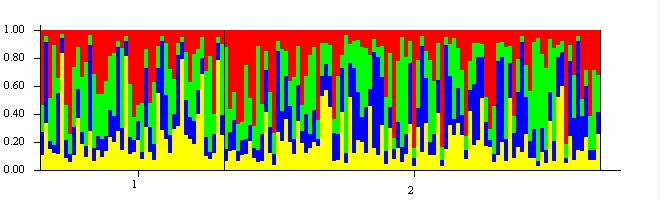** | 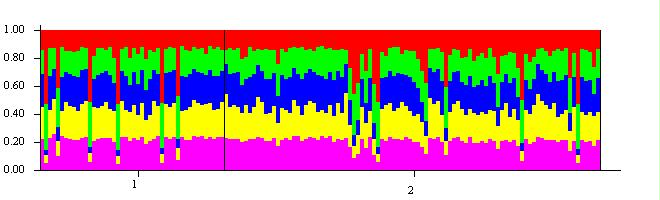 |
|  | BerbyelvaC | 94 | 2007-2008 |  |  |
|  |  | **3049** | **1971-2010** |  |  |
