# Supplementary material for: Three Decades of Farmed Escapees in the Wild: A Spatio-Temporal Analysis of Atlantic Salmon Population Genetic Structure throughout Norway
Source: PLoS One. 2012 Aug 15;7(8):e43129. doi: 10.1371/journal.pone.0043129 (PMC3419752; doi:10.1371/journal.pone.0043129)
Supplement: Figure S2 — Hierarchical Bayesian clustering of the 21 rivers in the historical and contemporary data sets. (DOC) [file pone.0043129.s002.doc]

**Three decades of farmed escapees in the wild: a spatio-temporal analysis of Atlantic salmon population genetic structure throughout Norway**

Kevin A. Glover^1*^, María Quintela^2^, Vidar Wennevik^1^, François Besnier^1^, Anne G. E. Sørvik^1^, Øystein Skaala^1^

**Fig. S2. Supporting information.**

**Hierarchical Bayesian clustering of the 21 rivers in the historic and contemporary data sets computed for total (a) and neutral loci (b).**

**(a)**

**
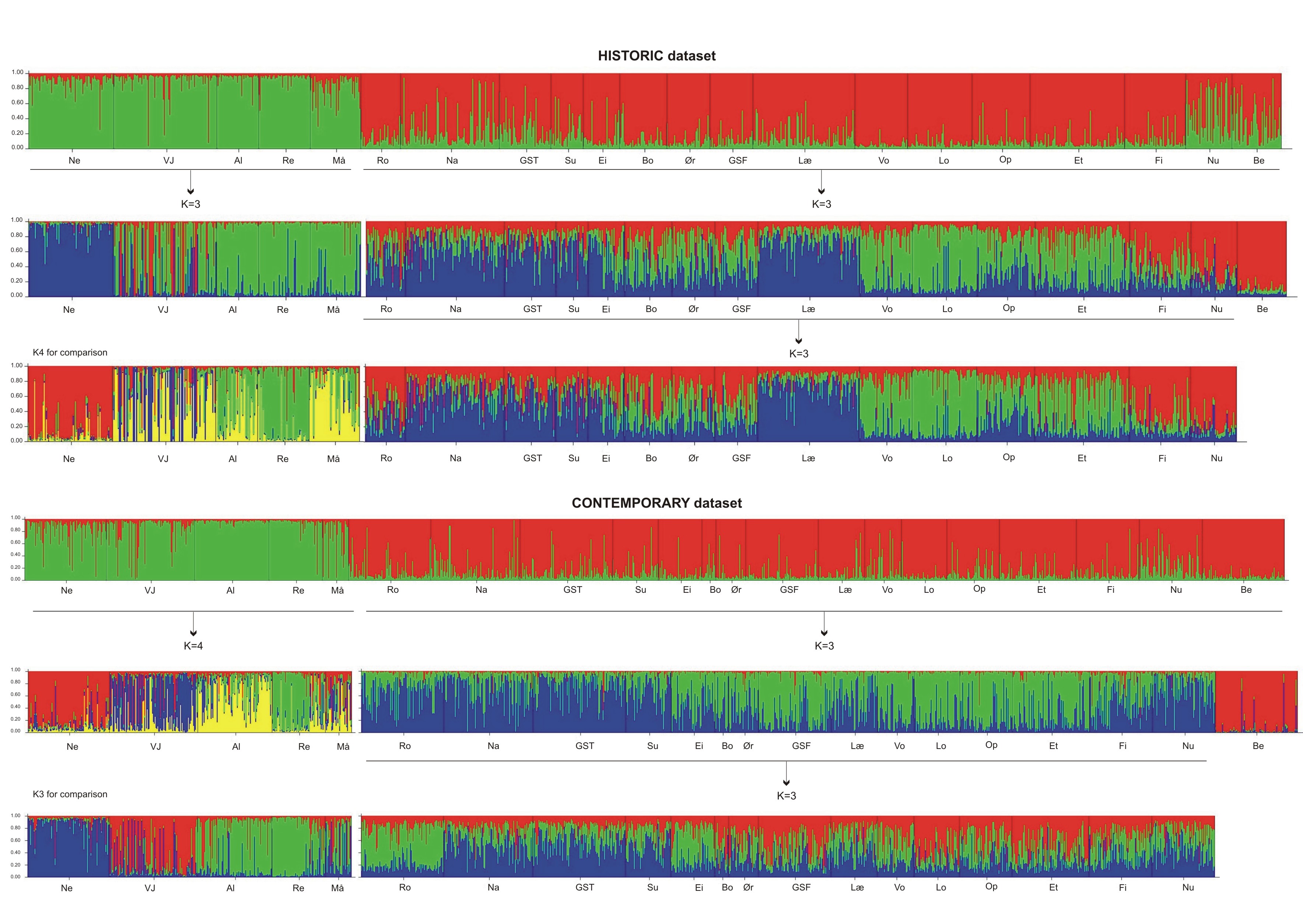
**

**(b)**

**
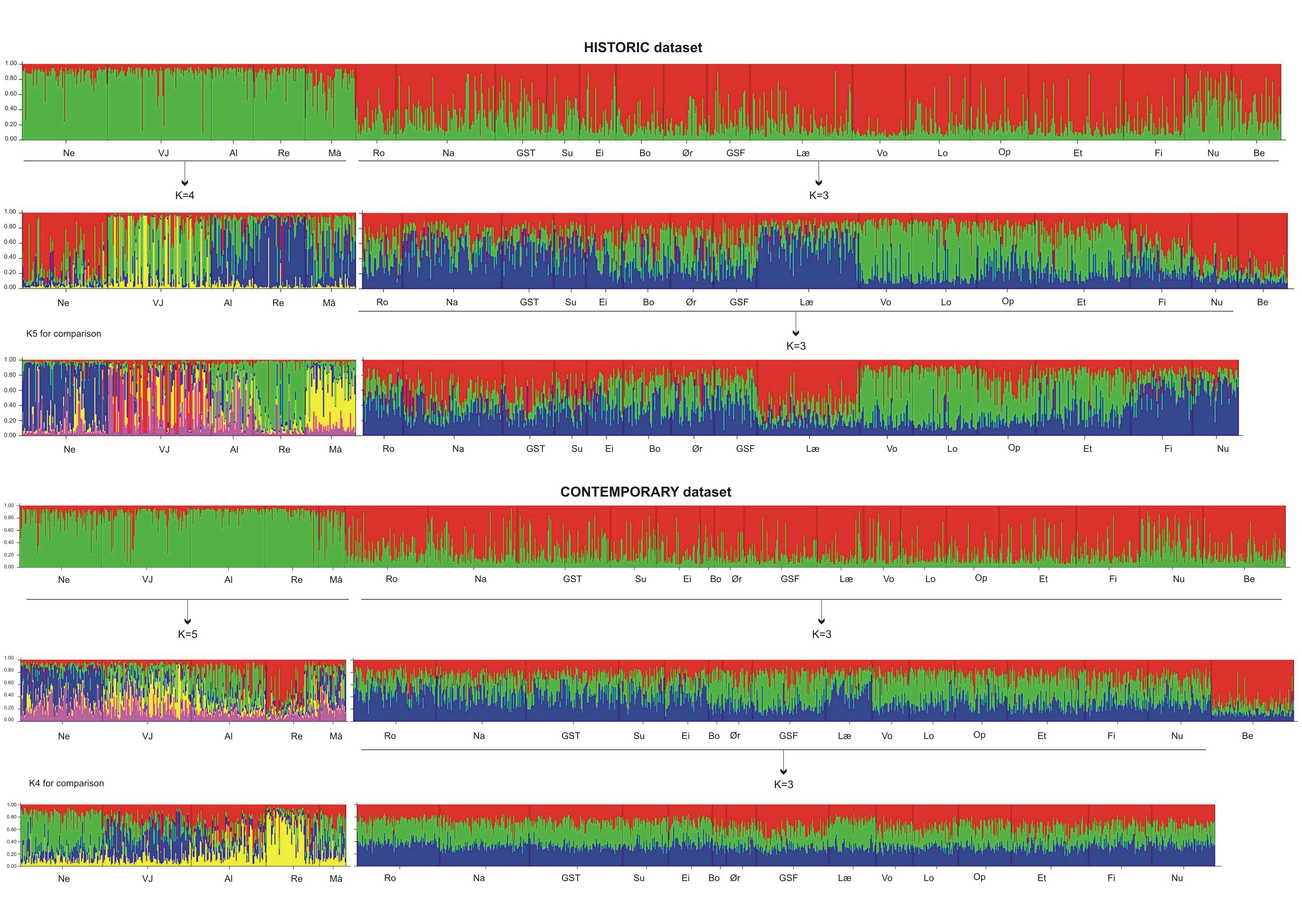
**
