# Supplementary material for: Three Decades of Farmed Escapees in the Wild: A Spatio-Temporal Analysis of Atlantic Salmon Population Genetic Structure throughout Norway
Source: PLoS One. 2012 Aug 15;7(8):e43129. doi: 10.1371/journal.pone.0043129 (PMC3419752; doi:10.1371/journal.pone.0043129)

**Three decades of farmed escapees in the wild: a spatio-temporal analysis of Atlantic salmon population genetic structure throughout Norway**

Kevin A. Glover^1*^, María Quintela^2^, Vidar Wennevik^1^, François Besnier^1^, Anne G. E. Sørvik^1^, Øystein Skaala^1^

**Fig. S3. Supporting information.**

**Admixture analysis of 21 Atlantic salmon populations distributed throughout Norway including samples from 9 distinct farm sources.**

Analyses are split into northern (5 wild populations and 9 farms) and southern (16 wild population and 9 farms) clusters due to a deep evolutionary divide. See Table 1 in manuscript for sample names. Analyses conducted both with and without prior for the farmed strains.


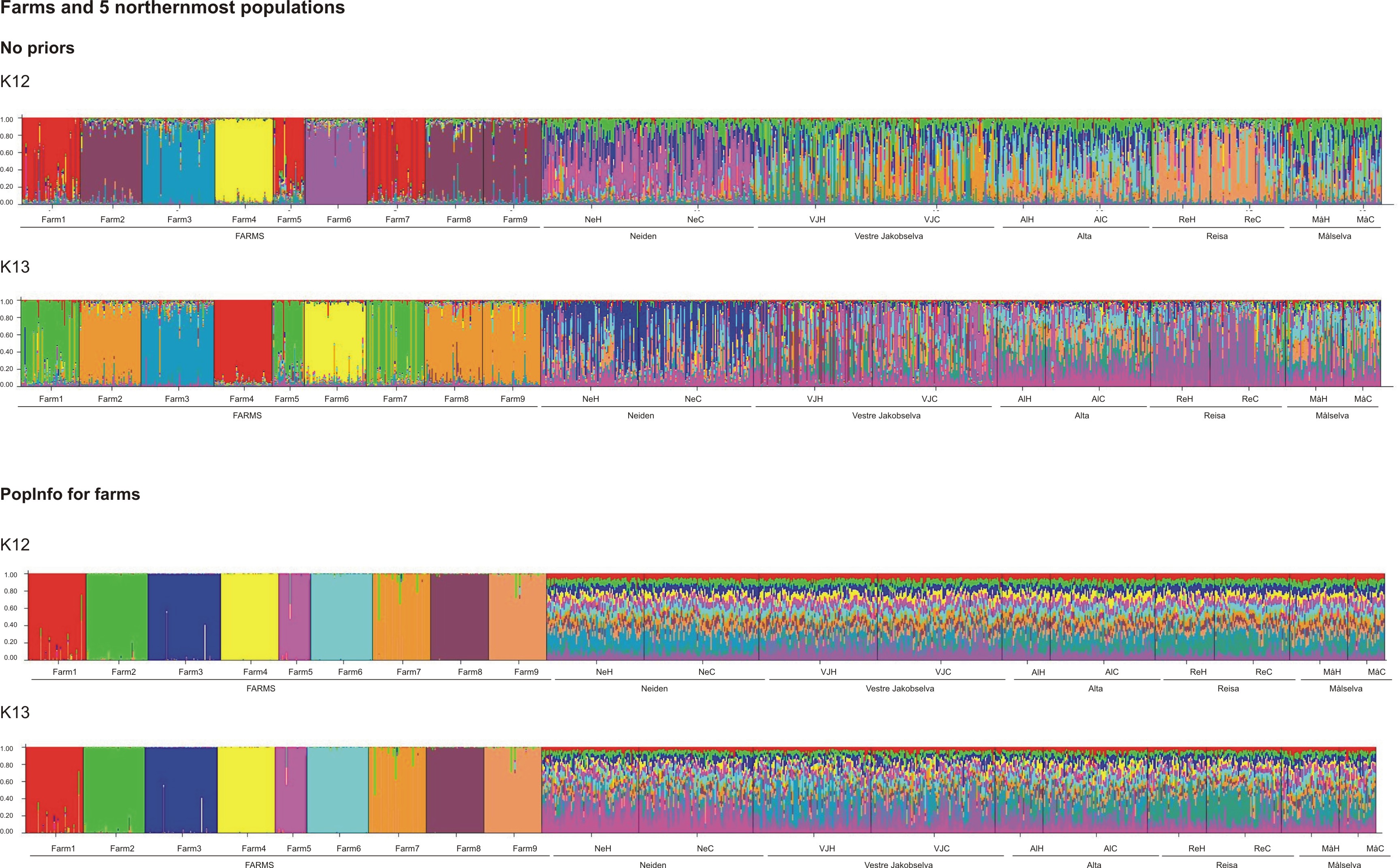


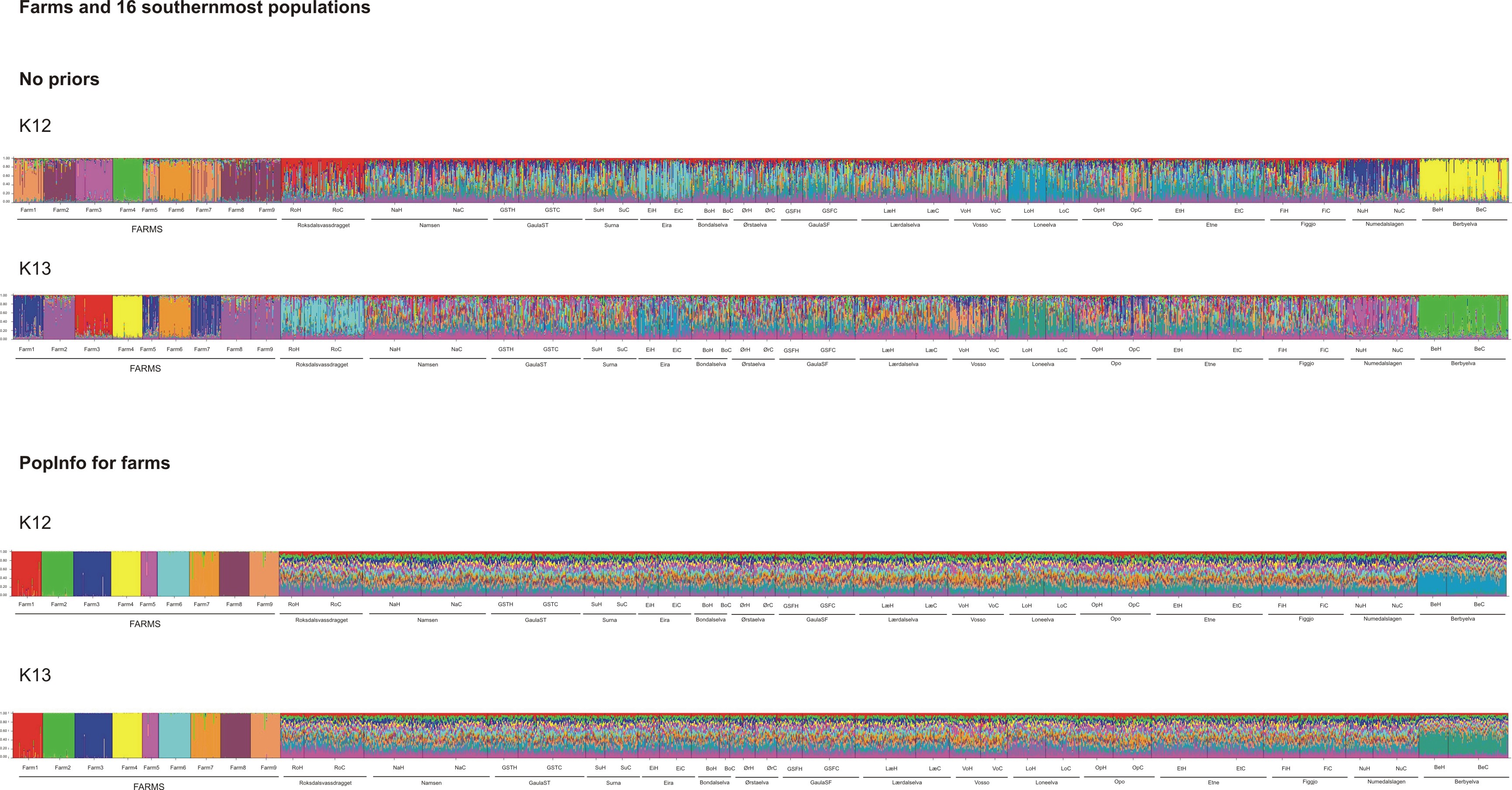

Supplement: Figure S3 — Bayesian clustering of the 21 rivers in the historical and contemporary data sets when combined together with data from 9 distinct farm sources. (DOC) [file pone.0043129.s003.doc]
