# Supplementary material for: Three Decades of Farmed Escapees in the Wild: A Spatio-Temporal Analysis of Atlantic Salmon Population Genetic Structure throughout Norway
Source: PLoS One. 2012 Aug 15;7(8):e43129. doi: 10.1371/journal.pone.0043129 (PMC3419752; doi:10.1371/journal.pone.0043129)
Supplement: Text S1 — Description of methods and results for identification of markers putatively under selection. (DOC) [file pone.0043129.s008.doc]

**Text S1 - SUPPORTING INFORMATION**

**Three decades of farmed escapees in the wild: a spatio-temporal analysis of Atlantic salmon population genetic structure throughout Norway**

Kevin A. Glover^1*^, María Quintela^2^, Vidar Wennevik^1^, François Besnier^1^, Anne G. E. Sørvik^1^, Øystein Skaala^1^

**MATERIAL AND METHODS**

**Detecting loci under selection**

A growing number of statistical approaches are available to identify putative non-neutral loci (*e.g.* Joost *et al*. (2007)). To minimize the risk of detecting false positives, we used two different approaches to detect loci with a pattern deviating from neutrality. First, we used the hierarchical Bayesian method described in Beaumont, Balding (2004) as implemented in BayeScan software (Foll, Gaggiotti, 2008). This method is based on the principle that genetic differentiation among populations living in contrasting environments is expected to be different for loci under selection than for the rest of the genome. BayeScan estimates population-specific F_ST_ coefficients and uses a cut-off based on the mode of the posterior distribution (Foll, Gaggiotti, 2008). Due to assumptions and algorithms that avoid the detection of false positives, outliers detected by BayeScan are likely to be good candidate adaptive regions of the genome (Par*is et a*l., 2010; Pérez-Figuer*oa et a*l., 2010). BayeScan is based on a logistic regression model in which each logit value of genetic differentiation F_ST_ (*i*, *j*) for locus *i* in population *j* is decomposed as a linear combination of the coefficients of the logistic regression, *α_i_* and *β_j_*, corresponding, respectively, to a locus effect and to a population effect. The posterior probability of locus *i* being under selection is estimated by defining two alternative models, one that includes *α_i_* and another that excludes it. To identify loci under selection, we focused on the posterior distribution of *α_i_*; thus, a positive value suggests that locus *i* is subject to directional selection, whereas a negative value suggests that stabilizing selection is tending to homogenize allele frequencies over the populations. The respective posterior probabilities of these two models are estimated using a reversible jump Markov chain Monte Carlo (RJMCMC) approach. The posterior probability that a locus is subjected to selection (P (*α_i_* ≠ 0)), is then estimated from the output of the RJMCMC by counting the number of times *α_i_* is included in the model. The estimation of model parameters was automatically tuned on the basis of short pilot runs (10 pilot runs, length 10000, burn in 100000). The sample size was set to 10000 and the thinning interval to 50 as suggested by Foll and Gaggiotti (2008), resulting in a total chain length of 600000 iterations. The Bayes factor (BF) criteria defined by Jeffreys (1961) as the ratio of the probabilities of the data under alternative hypotheses (selection *vs*. no selection) was used to consider a locus under selection. Loci were ranked according to their estimated posterior probability and those with a posterior probability over 0.99, were retained as outliers, which corresponds to a Bayes Factor >2 (*i.e.* “decisive selection” (Foll, Gaggiotti, 2006)) and provides substantial support for accepting the model.

Secondly, we used the Beaumont, Nichols (1996) Fdist approach, implemented in LOSITAN (Ant*ao et a*l., 2008) selection detection workbench for codominant markers. LOSITAN uses coalescent simulations to generate a null distribution of F_ST_ values based on an infinite island model for the populations and an infinite allele model or a stepwise mutation model for polymorphism. Loci with an unusually high F_ST_ are putatively under directional selection, while loci with low F_ST_ value are considered to be potentially under stabilizing selection. In this study, we simulated the neutral distribution of F_ST_ with 1000000 iterations at a significance *P* value of 0.005. Runs were performed using the two possible mutation models: the stepwise mutation model and the infinite allele model. This method also implements a multitest correction based on false discovery rates (FDR) that is fundamental to avoid high overestimation of the percentage of outliers (e.g. 1% of false positive with a threshold of 99%).

**RESULTS**

When combining the results obtained from both analytical approaches (Table A1, Table A2) we found that samples corresponding to the historic data set showed three loci under directional selection and five under stabilizing selection whereas the contemporary data set showed the same loci under directional selection but only two of the former ones under stabilizing selection. Interestingly, loci SSsp2216, Ssa197, and SsaD144 that had been depicted under stabilizing selection in the historic data set became neutrals in the contemporary samples.

The neutral data set was, therefore, built after excluding the eight loci that showed patterns deviating from neutrality; *i.e.* MHC2, SsaF43, Ssa289, SSsp2216, Ssa197, SsaD157, SsaD144, SSsp2201.

**REFERENCES**

Antao T, Lopes A, Lopes R, Beja-Pereira A, Luikart G (2008) LOSITAN: A workbench to detect molecular adaptation based on a F_ST_-outlier method. *BMC Bioinformatics* **9**, 323.

Beaumont M, Nichols R (1996) Evaluating loci for use in the genetic analysis of population structure. *Proceedings: Biological Sciences* **263**, 1619-1626.

Beaumont MA, Balding DJ (2004) Identifying adaptive genetic divergence among populations from genome scans. *Molecular Ecology* **13**, 969-980.

Foll M, Beaumont MA, Gaggiotti O (2008) An approximate Bayesian computation approach to overcome biases that arise when using Amplified Fragment Length Polymorphism markers to study population structure. *Genetics* **179**, 927-939.

Foll M, Gaggiotti O (2006) Identifying the environmental factors that determine the genetic structure of populations. *Genetics* **174**, 875-891.

Foll M, Gaggiotti O (2008) A genome-scan method to identify selected loci appropriate for both dominant and codominant markers: A Bayesian perspective. *Genetics* **180**, 977-993.

Jeffreys H (1961) *Theory of Probability*, 3rd edn. Clarendon Press, Oxford, UK.

Joost S, Bonin A-, Bruford MW*, et al.* (2007) A spatial analysis method (SAM) to detect candidate loci for selection: towards a landscape genomics approach to adaptation. *Molecular Ecology* **16**, 3955-3969.

Paris M, Boyer S, Bonin A*, et al.* (2010) Genome scan in the mosquito *Aedes rusticus*: population structure and detection of positive selection after insecticide treatment. *Molecular Ecology* **19**, 325-337.

Pérez-Figueroa A, García-Pereira MJ, Saura M, Rolán-Álvarez E, Caballero A (2010) Comparing three different methods to detect selective loci using dominant markers. *Journal of Evolutionary Biology* **23**, 2267-2276.

**Table A1.-** **Historic samples**.- Shaded cells with numbers in bold depict loci detected to be under selection by: BayeScan (log_10_(BF)>2 corresponding to “decisive selection”) and LOSITAN at a significance *P* value of 0.01 under the stepwise and infinite alleles mutation models. Note that locus SsaF43 with log_10_(BF)=1.79 is above the threshold of “very strong selection”

| **Locus** | **LOSITAN** | | **BayeScan** | | **Selection** |
| --- | --- | --- | --- | --- | --- |
|  | **Stepwise** | **Infinite alleles** |  |  |  |
|  | **P (Simul F_ST_ <sample F_ST_)** | **P (Simul F_ST_ <sample F_ST_)** | **log_10_(BF)** | **F_ST_** |  |
| **MHC2** | 0.9997 | 0.9994 | 1000 | 0.07074 | Directional |
| **SsaF43** | 0.9995 | 0.9998 | 1.7944 | 0.065242 | Directional |
| **Ssa289** | 0.9947 | 0.9985 | 2.8232 | 0.071331 | Directional |
| **SSsp2216** | 0.0004 | 0.0000 | 2.1216 | 0.020748 | Stabilizing |
| **Ssa197** | 0.0060 | 0.0000 | 2.2902 | 0.020304 | Stabilizing |
| **SsaD157** | 0.0042 | 0.0000 | 2.2988 | 0.020314 | Stabilizing |
| **SsaD144** | 0.0176 | 0.0000 | 3.5227 | 0.017856 | Stabilizing |
| **SSsp2201** | 0.0160 | 0.0000 | 1000 | 0.015246 | Stabilizing |

**Table A2.-** **Contemporary samples**.- Shaded cells with numbers in bold depict loci detected to be under selection by: BayeScan (log_10_(BF)>2 corresponding to “decisive selection”) and LOSITAN at a significance *P* value of 0.01 under the stepwise and infinite alleles mutation models.

| **Locus** | **LOSITAN** | | **BayeScan** | | **Selection** |
| --- | --- | --- | --- | --- | --- |
|  | **Stepwise** | **Infinite alleles** |  |  |  |
|  | **P (Simul F_ST_ <sample F_ST_)** | **P (Simul F_ST_ <sample F_ST_)** | **log_10_(BF)** | **F_ST_** |  |
| **MHC2** | 0.9999 | 1.0000 | 3.9999 | 0.048866 | Directional |
| **Ssa289** | 0.9968 | 0.9894 | 3.0965 | 0.050822 | Directional |
| **SsaF43** | 0.9998 | 1.0000 | 0.84465 | 0.041503 | Directional |
| **SsaD157** | 0.0186 | 0.0002 | 3.6988 | 0.011784 | Stabilizing |
| **SSsp2201** | 0.0211 | 0.0000 | 2.35459 | 0.013147 | Stabilizing |
